# Characterization of Bacterial Communities in Selected Smokeless Tobacco Products Using 16S rDNA Analysis

**DOI:** 10.1371/journal.pone.0146939

**Published:** 2016-01-19

**Authors:** Robert E. Tyx, Stephen B. Stanfill, Lisa M. Keong, Angel J. Rivera, Glen A. Satten, Clifford H. Watson

**Affiliations:** 1 Division of Laboratory Sciences at the Centers for Disease Control and Prevention, Atlanta, GA, United States of America; 2 Division of Reproductive Health, Center for Disease Control and Prevention, Atlanta, GA, United States of America; 3 Oak Ridge Institute of Science and Education, Oak Ridge, TN, United States of America; 4 Battelle Analytical Services, Atlanta, GA, United States of America; University Paris South, FRANCE

## Abstract

The bacterial communities present in smokeless tobacco (ST) products have not previously reported. In this study, we used Next Generation Sequencing to study the bacteria present in U.S.-made dry snuff, moist snuff and Sudanese toombak. Sample diversity and taxonomic abundances were investigated in these products. A total of 33 bacterial families from four phyla, Actinobacteria, Firmicutes, Proteobacteria and Bacteroidetes, were identified. U.S.-produced dry snuff products contained a diverse distribution of all four phyla. Moist snuff products were dominated by Firmicutes. Toombak samples contained mainly Actinobacteria and Firmicutes (*Aerococcaceae*, *Enterococcaceae*, *and Staphylococcaceae*). The program PICRUSt (Phylogenetic Investigation of Communities by Reconstruction of Unobserved States) was used to impute the prevalence of genes encoding selected bacterial toxins, antibiotic resistance genes and other pro-inflammatory molecules. PICRUSt also predicted the presence of specific nitrate reductase genes, whose products can contribute to the formation of carcinogenic nitrosamines. Characterization of microbial community abundances and their associated genomes gives us an indication of the presence or absence of pathways of interest and can be used as a foundation for further investigation into the unique microbiological and chemical environments of smokeless tobacco products.

## Introduction

It is estimated that more than 300 million people worldwide use some form of smokeless tobacco (ST). Cancer, heart disease, diabetes and other health effects are linked to ST use [1]. Past studies of health effects associated with ST have focused primarily on chemical constituents, including addictive, toxic, and carcinogenic compounds absorbed during use [2–4], even though microorganisms have been found in tobacco or tobacco products [5–7].

To date, a survey of bacteria present in smokeless tobacco has not been reported, even though bacteria are known to generate toxins, pro-inflammatory biomolecules (flagellin, LPS, lipid A) [8,9], or generate nitrite, a key precursor in the formation of tobacco-specific *N*’-nitrosamines (TSNAs), the most abundant carcinogens in many ST products [10,11]. Products made using fermentation, such as toombak, moist and dry snuff, and cigar tobacco, generally contain higher levels of certain carcinogens (i.e. nitrosamines) than unfermented products [12–16]. Modifications to processing and storage conditions, especially the use of pasteurization in Swedish-made snus, that lowers microbial levels, tend to decrease levels of harmful constituents in tobacco products [9,17–20]. These findings suggest characterizing bacteria in ST products may be an important step towards understanding product microbiology with the goal of reducing the levels of certain harmful compounds in ST products.

Previous studies of microbes in tobacco or tobacco products have relied heavily on culture-based approaches that likely under-represent community diversity. Only a few studies have used culture-independent methods, including two 16S rDNA analyses of flue-cured tobacco leaves [21,22], a study on cigarette tobacco [6], and a detailed study of fermented cigar tobacco [5].

This report represents an initial survey of bacterial communities using 16S rDNA, along with an imputed metagenomics analysis, to better understand the specific nitrogen pathways and genes that encode bacterial toxins, pro-inflammatory biomolecules, or those that confer antibiotic or drug resistance that may be present in ST products.

## Materials and Methods

### DNA Extraction of Tobacco Samples

U.S. domestic tobacco products (dry and moist snuff) and Swedish-made snus were obtained by an outside contractor at retail sites in the Atlanta area, undisclosed to researchers. Sudanese toombak samples were graciously provided by Ghazi Zaatari, M.D. (Department of Pathology and Laboratory Medicine American University of Beirut; Beirut, Lebanon) who obtained them from stores in Khartoum, Sudan. All samples received were barcoded and stored at -20°C. Samples were thawed to room temperature and 200 mg of ST was measured for DNA extraction and purification. An enzyme cocktail was prepared by mixing 5 μl lysostaphin (5 μg/μl), 5 μl lysozyme (10 μg/μl), and 15 μl mutanolysin (1 μg/μl). Separately, the measured smokeless tobacco samples and 1000 μl molecular grade 1X phosphate buffered saline (PBS) were added to Lysing Matrix J bead-beating tubes (MP Biomedicals, Santa Ana, CA, USA). The 25 μl aliquot of the enzyme cocktail was added to the bead-beating tubes and the resulting mixture incubated at 37°C for 30 minutes. Immediately upon incubation completion, 10 μl Proteinase K (20 μg/μl) and 50 μl 10% SDS were added to the tubes and incubated at 55°C for 30 minutes. All reagents mentioned above were obtained from Sigma-Aldrich (St. Louis, MO, USA). Following incubation, tubes were bead-beaten for 2 minutes at 4800 RPM in a Mini-BeadBeater (BioSpec, Inc.; Bartlesville, OK, USA). Sample tubes were centrifuged at 10,000 X *g* for 5 minutes, and the supernatant was transferred to a new tube. Each sample was then diluted 2:1 with 100% ethanol. Binding, washing and elution of DNA was accomplished using QIAamp Mini Spin columns (Qiagen Sciences Inc.; Germantown, MD, USA) as specified by the manufacturer. Best results were obtained when DNA was passed through a second QIAamp Mini Spin column. Final DNA concentrations, after the second filtration, ranged from 0.5 − 11.5 ng/μl for all domestic snuff and, 32.6 − 48.5 ng/μl for the toombak samples. The extraction method described above was also performed on two Swedish-made snus products; however, no DNA was quantified above the detection limit (0.01 ng/μl). Furthermore, PCR amplification of these extractions did not produce detectable amplicons.

### 16S Amplification and Creation of DNA libraries

PCR was performed in a Biorad C1000 Touch Thermal Cycler (Hercules, CA) with PCR reactions using Q5 Hot Start Supermix (New England Biolabs, Ipswich, MA, USA) with a primer concentration of 0.5 μM of each primer. The PCR parameters were as follows: A denaturation step of 98°C for 30 seconds, followed by 30 cycles of a melting step at 98°C for 10 seconds, an annealing step of 55°C for 20 seconds, an extension step of 72°C for 25 seconds, followed by a final extension step at 72°C for 5 minutes. Resulting amplicons were visualized on 1% agarose gels to check for amplification efficiency and size. Amplicons were purified using AMPure XP magnetic beads (Beckman Coulter, Brea, CA, USA), run on an Invitrogen Size-Select 2% E-gel (Thermo Fisher Scientific Inc.; Waltham, MA, USA) and the appropriate band extracted from the gel, per the manufacturer’s protocol. Nucleic acids were quantified using a dsDNA High Sensitivity Assay and measured with a Qubit 2.0 fluorometer (Life Technologies/Thermo Fisher Scientific Inc.; Waltham, MA, USA). Qubit values were used to calculate the molar concentrations of each amplicon library and to perform the appropriate dilutions. Equimolar pools of amplicon libraries were prepared with 15–18 libraries per sequencing run.

The V4 hyper-variable region of the 16S rDNA (approximately 290 base pairs in length) was PCR amplified using barcoded fusion primers with a few degenerate base pairs as described by Bokulich *et al*., [23] (S1 Table). The V4 region primer set was chosen to obtain the best coverage of most environmental organisms [24]. The forward primer was expected to cover 50.6% of all annotated archaeal sequences and 79.1% of annotated bacterial sequences with no mismatch based on the Silva TestProbe against the Silva Ref NR data set as of 1/29/2015 [25–27]. The reverse primer was expected to cover 77.4% of archaeal sequences and 76.4% of bacterial sequences with no mismatches. These primers, as a set, are expected to cover 46.2% of archaea and 72.3% of bacteria with no mismatches. The phyla with the least coverage by this primer set are mainly unculturable and/or candidate phyla, including Caldiserica (1.8% sequences of this candidate phylum covered), CD12 (2.6%), Chlamydiae (0.7%), FBP (12.7%), OD1 (6.0%), OP11 (1.4%), TM7 (3.5%).

### Next Generation Sequencing

Libraries were sequenced using the Ion Torrent PGM (Thermo Fisher Scientific Inc.; Waltham, MA). Templating was performed using the Ion PGM Template OT2 400 Kit on the Ion OneTouch 2 System. Enrichment was performed using the Ion OneTouch ES system. Sequencing was carried out using 316 V2 chips and the Ion PGM Sequencing 400 Kit. Data was output to a fastq file, which was then transferred from the Ion Torrent Server computer to a dedicated bioinformatics workstation for further analysis. Raw data files of three sequencing runs were combined into a file containing 8,821,880 sequences (S2 Table). Raw sequence data was submitted to NCBI Short Read Archive (SRA) under accession number **SRR2157163**.

### Bioinformatic Analysis

The FastX toolkit (from the Gregory J. Hannon Lab, Cold Spring Harbor, NY, http://cancan.cshl.edu/labmembers/gordon/fastx_toolkit/index.html) was used to trim the sequences (see Supplemental Bioinformatics section for more detail). QIIME version 1.8.0 was used to assign reads to operational taxonomic units (OTUs) and analyze alpha and beta-diversities, and relative abundances of taxonomic groups [28]. Raw read numbers from the script that splits output based on barcode sequence are given in table format in S1 Fig. 50 OTUs (0.92% of OTUs) corresponding to chloroplast and mitochondria (10.7% and 0.25% of total reads, respectively) were manually trimmed from the OTU table to prevent interference with analysis of microbial communities. OTUs that failed alignment (29 OTUs, representing 0.54% of the remaining OTUs, corresponding to 9056 reads of the remaining 3,747,634) were also trimmed, as they would not be classifiable in the tree file and could also negatively impact the imputed metagenomics analysis.

The OTU table was further trimmed to remove low-frequency OTUs through a custom R function (*threshold.matrix.R—see* S1 Text). OTUs not having at least 0.1% abundance in some product were removed from the OTU table. Cells containing less than 0.1% of overall abundance were also set to zero. Using this threshold, 190,398 of 3,738,578 reads (5.09%) were trimmed, while the total number of OTUs decreased from 5345 to 235 (95.6% removed). Removing these sparse OTUs allowed us to concentrate our analysis on the OTUs that contain the bulk (94.9%) of reads, while removing the OTUs that are the most difficult to compare across products due to differences in library size.

Within-sample (alpha) diversity and alpha-rarefaction plots were generated in QIIME using various metrics. Distances between samples (beta diversity) were calculated by QIIME using the weighted UniFrac distance. Both calculations used only reads from the trimmed OTU table. Library sizes were rarefied to the minimum number of reads of a single product in the OTU table before calculating the beta diversity. S1 Text contains further detail of bioinformatics analysis.

### Imputed Metagenomics Analysis and Gene Ontology Phylogenetic Investigation of Communities by Reconstruction of Unobserved States (PICRUSt)

Copy number normalization of 16S for each OTU was calculated using the PICRUSt script *normalize_by_copy_number*.*py* [29,30], which encompasses data from the United States Department of Energy Joint Genomic Institute’s Integrated Microbial Genomes (IMG) database. Functionality, as represented by KEGG Orthology [31–33] annotation, was predicted using the PICRUSt scripts (*predict_metagenomes*.*py* and *metagenome_contributions*.*py*). Due to the varying read numbers per product, PICRUSt output representations were adjusted (normalized) based on PICRUSt occurences per 100,000 reads.

### Statistical Analysis

Analysis of Variance (ANOVA) was used to determine percent differences between products. The Adonis function in the R package Vegan was used to partition the (weighted) UniFrac distance matrix to obtain the proportion of variation attributed to type and specimen [34].

A multivariate logistic prediction model was used to discern which OTUs discriminate type. The R package “glmnet” fits a polytomous logistic regression model with a regularization penalty. We used a regularization penalty that was equal mixture of Lasso and ridge regression penalties (alpha = 0.5 in glmnet), as this value tends to include groups of correlated variables [35]. Using leave-one-out cross-validation, glmnet selected the penalty parameter to be the largest value that provided perfect classification. Before fitting the model, we converted the data matrix from counts to proportions and centered rows and columns, but did not scale the variables so as to favor higher-prevalence species during model selection (we also used the scale = FALSE option in glmnet to preserve the original scale).

## Results and Discussion

### Bacterial Microbiome Sequencing

Using high-throughput sequencing of the 16S rDNA, we analyzed the constituency of the microbial communities in 15 smokeless tobacco products, including six U.S. dry snuffs, seven U.S. moist snuffs, and two Sudanese toombak samples.

**Fig 1:**
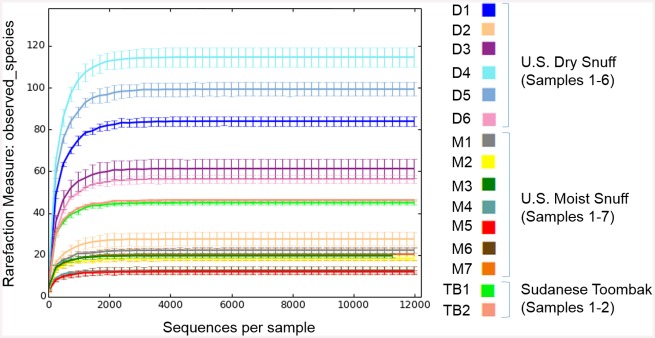
Alpha-diversity measure using QIIME. Plots shown represent estimated OTU abundance in increasingly rarefied subsamples (n = 50) of the original data set. Error bars represent standard deviation. Alpha-rarefaction plots were generated in QIIME using the observed_species metric for estimating alpha (within-sample) diversity.

Alpha-diversity rarefaction plot results suggest that with a 0.1% abundance threshold at the OTU level, even the samples with lowest read depths were sufficiently well sampled to give an acceptable measure of species diversity (Fig 1). In fact, we observed saturation of species diversity in the rarefaction curves for all of the samples examined, using several different metrics for measuring alpha diversity (Fig 1, S2 Fig). Interestingly, dry snuff samples (D1 –D6, Fig 1), which might be expected to have lower diversities based on the lower relative moisture content of these samples, exhibited higher overall species diversity than the moist snuff (M1 –M7, Fig 1) and toombak samples (TB1, TB2, Fig 1, S2 Fig).

**Fig 2:**
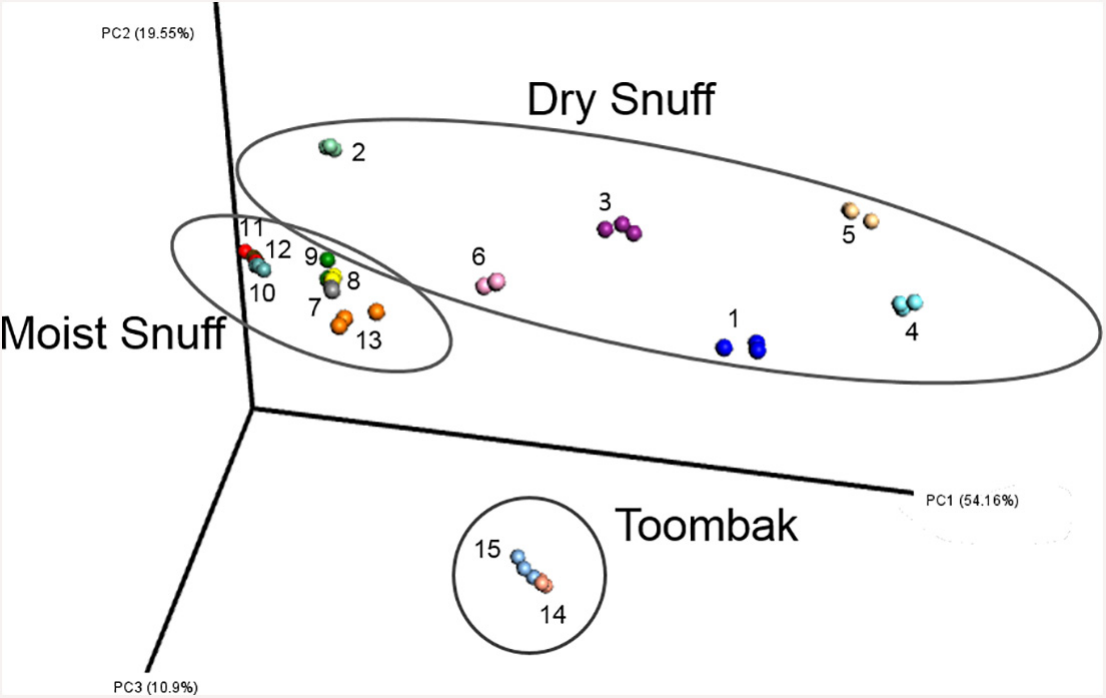
3-Dimensional Weighted Unifrac Principal Component Analysis showing replicate, sample and type variability. Legend: 1: D1 2: D2 3: D3 4: D4 5: D5 6: D6 7: M1 8: M2 9: M3 10: M4 11: M5 12: M6 13: M7 14: TB1 15: TB2. Weighted UniFrac distances were used to ordinate the samples, allowing visualization of within and between replicate variability.

A distance matrix was constructed using Weighted UniFrac distances to assess beta (between-product) diversity. Principal components analysis of the distance matrix demonstrated that distances between replicates of the same product were notably smaller than distances between products (Fig 2, S3 Fig). Statistical analysis using ANOVA confirmed that type (moist, dry, or toombak) accounted for 54.8% of the total sum of squared distances, while differences within ST type (between-product variation) accounted for 44.8% of variation. The residual variation (between-replicate variation) accounted for less than 1% of variation (Table 1). Microbial community compositions were determined to be significantly different between product types tested (dry vs moist vs toombak, p<10^−6^) (Table 1).

**Table 1:** Statistical Analysis of within and between-sample differences based on Analysis of Variances (ANOVA).

Variable	Df	Sum of Squares	Mean Squares	F statistic	R^2^	% Variability	Statistical Significance
Product Types	2	3.2628	1.63139	1788.28	0.54758	54.76	***
Products	12	2.6684	0.22237	234.75	0.44783	44.78	***
Replicates	30	0.0274	0.00091		0.00459	0.46	
Total	44	5.9585			1.00000	100	

*** = p < 0.001

Analysis of Variance (ANOVA) of weighted UniFrac distance data. The F statistic determines whether the proportion of variability explained by a variable is significant. The R^2^ value is the estimated percent of total variability explained by each variable. Df is degrees of freedom.

### Relative abundance of microbial communities

**Fig 3:**
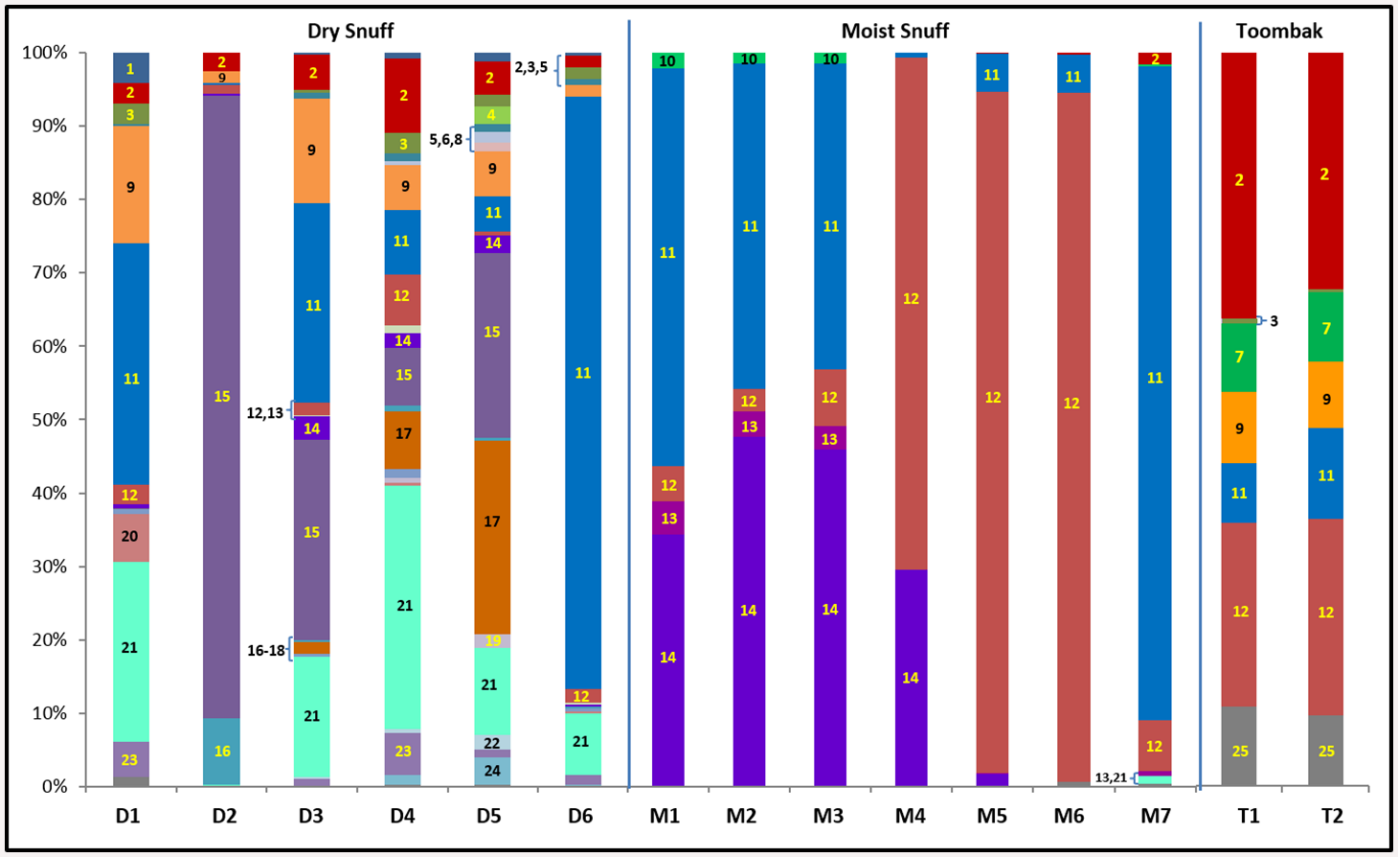
Relative abundance at family level of taxonomy. The relative abundances at the family level of taxonomic classification were calculated using QIIME. Each bacterial family is represented as a different color in the bar graphs below. Combined relative abundances total 100% for each individual product. Numbering is as follows: 1) *Brevibacteriaceae* 2) *Corynebacteriaceae* 3) *Dermabacteraceae* 4) *Microbacteriaceae* 5) *Micrococcaceae* 6) *Promicromonosporaceae* 7) *Yaniellaceae* 8) *Sphingobacteriaceae* 9) *Bacillaceae* 10) *Planococcaceae* 11) *Staphylococcaceae* 12) *Aerococcaceae* 13) *Carnobacteriaceae* 14) *Enterococcaceae* 15) *Lactobacillaceae* 16) *Leuconostocaceae* 17) *Acetobacteraceae* 18) *Alcaligenaceae* 19) *Comamonadaceae* 20) *Oxalobacteraceae* 21) *Enterobacteriaceae* 22) *Moraxellaceae* 23) *Pseudomonadaceae* 24) *Xanthomonadaceae* 25) All Others (*Bogoriellaceae*, *Flavobacteriaceae*, *Nocardiaceae*, *Aurantimonadaceae*, *Methylobacteriaceae*, *Rhizobiaceae*, *Alteromonadaceae*, *Halomonadaceae*, and *Rhodobacteraceae)*.

ST samples exhibited a wide range of taxonomic diversity at the family level. Overall, 33 bacterial families from four phyla, Actinobacteria (9 families), Firmicutes (8 families), Proteobacteria (14 families), and Bacteroidetes (2 families), were detected with an abundance of at least 0.1% in a sample. Dry snuff contained as few as 9 families (D2) to as many as 27 families (D4); whereas, moist snuff products ranged from 3 families (M4) to 8 families (M7). Toombak samples had 6 (TB1) to 7 (TB2) families, with ~10% of reads for each toombak sample remaining unclassified at the family level. Only the *Staphylococcaceae* and *Aerococcaceae* families were present at a 0.1% abundance or greater in all 15 products analyzed (Fig 3, S3 Table). Dry snuff products were significantly more diverse at the family level than moist snuff products, with means of 19 families per dry product vs 5 families per moist product (p = 0.0002).

Moist snuff contained a relatively low number of bacterial families, with all samples being overwhelmingly dominated (>95% of all reads in moist samples) by Firmicutes, including *Staphylococcaceae* (most abundant family in M1 and M7), *Aerococcaceae* (most abundant in M4, M5, and M6), and *Enterococcaceae* (most abundant in M2 and M3). Three of the moist snuff samples, M1, M2 and M3 had very similar bacterial community structures, dominated by *Enterococcaceae and Staphylococcaceae*, with lower percentages of *Aerococcaceae*, *Carnobacteriaceae*, *and Planococcaceae*. Conversely, samples M4, M5, and M6 had a very high proportion (>65% of reads) of *Aerococcaceae* (Fig 3, S3 Table); whereas, M7 had the highest percentage of *Staphylococcaceae* (>85%) among the moist snuff products. It should be noted that M1, M2, and M3 were made by the same manufacturer and may have been manufactured from similar sources of raw tobacco. Products M4 and M5 were made by another manufacturer; whereas, M6 and M7 were each made by different manufacturers. The similarities between moist products from the same manufacturer may reflect common tobacco sources or processing environments.

Dry snuff products had much greater family diversity, with communities consisting of several phyla: primarily Actinobacteria, Firmicutes, and Proteobacteria, with Bacteroidetes present only in D4 and D5 at low levels (Fig 3, S3 Table). In dry snuff, five bacterial families predominated in the products tested here: *Bacillaceae* (>10% of D1 and D3), *Staphylococcaceae* (>25% of D1, D3, and >75% of D6), *Lactobacillaceae* (>80% of D2, >20% of D3 and D5), *Acetobacteraceae* (>25% of D5), and *Enterobacteriaceae* (>20% of D1, and D4, >10% of D3 and D5). The most dominant family in any dry snuff product was *Lactobacillaceae* in product D2, where >80% of the total sequences were from that family (S3 Table). Several other families were found at a prevalence of 2–10% in a single dry snuff, these included: *Aerococcaceae*, *Bacillaceae*, *Brevibacteriaceae*, *Corynebacteriaceae*, *Dermabacteraceae*, *Enterococcaceae*, *Leuconostocaceae*, *Microbacteriaceae*, *Moraxellaceae*, *Pseudomonadaceae*, and *Xanthomonadaceae*.

Products D1 and D2 were made by one manufacturer; whereas, D3, D4, and D5 were produced by another manufacturer. A single product, D6, from a third manufacturer was also analyzed. Unlike the moist product samples, dry snuffs did not exhibit similarity when comparing products from the same manufacturing company.

Brown toombak, a Sudanese cottage-industry product, was more similar to moist snuff, as both contained high relative abundances of *Staphylococcaceae* (8.1% and 12.3% in samples TB1 and TB2, respectively) and *Aerococcaceae* (25.1% and 26.8%), and low alpha diversity compared with dry snuff products. In contrast to moist domestic snuff, toombak contained a high percentage (>30% of all reads for each sample) of Actinobacteria (mainly *Corynebacteriaceae*) (Fig 3, S3 Table). Toombak samples contained only four other families, including *Dermabacteraceae*, *Yaniellaceae*, *Bacillacea*e and *Planococcaceae* (Fig 3, S3 Table).

Many of the bacterial genera identified in this study, including the *Bacillus*, *Corynebacterium*, *Staphylococcus*, *Pseudomonas*, and *Tetragenococcus*, have been identified previously in tobacco, and are primarily soil-borne or plant-associated; some of these groups also contain opportunistic pathogens [5,6]. Microbial species diversity in ST products is likely affected by a combination of factors. These factors include the endogenous and predominant soil bacteria in tobacco fields, parent populations in the tobacco seeds/seedlings, human-associated microbes introduced at harvesting and preproduction, resident microbial populations in processing environments (including curing and aging facilities), bacterial populations resident in fermentation vats, and fermentation bacteria added by the manufacturer, if any.

There are other factors that may affect diversity itself, or the sampling of diversity using isolated DNA. Physical parameters such as particle size (dry snuff is ground to a loose powder) and moisture content may impact the efficiency of the DNA extraction procedure on particular microbes. Moisture content of products (>50% moisture by weight for moist snuff vs ~6–7% moisture by weight for dry snuff) [13], could potentially affect the stability of DNA. If this is the case, dry products may allow a more comprehensive “view” of past microbial constituency compared with moist snuff products. Sampling of bacterial diversity could also be impacted (positively or negatively) by tobacco constituents or additives.

Although we have centered our discussion on families, in many cases, OTUs could be resolved to the genus or species level. The full OTU table and assigned taxonomy are given in S4 and S9 Tables, **respectively**. Bacteria identified in the study represent those found in this limited set of products and should not be taken as evidence that all products will contain the same types of bacteria.

### Using OTUs to Discriminate Types

To determine which OTUs discriminate the three tobacco types, we fit a multivariate logistic prediction model. This statistical method allows us to determine which taxonomic groups are characteristic of which product type. We used a prediction model with cross-validation so that the associations we report are likely not just the result of overfitting, but could reasonably be expected to be seen in future samples.

Nineteen OTUs were selected along with the coefficients for each OTU in the prediction model for each tobacco type (Table 2).

**Table 2:** Predictive Logistical Discrimination of OTU/taxonomy vs Product Type.

Phylum	Family	OTU assignment *	Dry	Moist	Toombak	OTU #
Actinobacteria	*Corynebacteriaceae*	*Corynebacterium stationis*	-1.63	-0.85	3.48	650615
Actinobacteria	*Corynebacteriaceae*	*Corynebacterium sp*.	-2.78	-1.34	5.12	810425
Actinobacteria	*Yaniellaceae*	*Yaniella sp*.	-0.95	0.00	1.95	115315
Actinobacteria	*Yaniellaceae*	*Yaniella sp*.	-0.83	0.00	1.74	790972
Firmicutes	*Bacillaceae*	*Bacillus sp*.	0.24	0.00	0.00	4297253
Firmicutes	*Bacillaceae*	n.d.	-0.31	0.00	1.01	3969822
Firmicutes	*Bacillaceae*	n.d.	0.13	0.00	0.00	1126217
Firmicutes	*Bacillaceae*	n.d.	0.15	0.00	0.00	4367385
Firmicutes	*Staphylococcaceae*	*Staphylococcus succinus*	-0.01	3.35	-2.34	4312974
Firmicutes	*Aerococcaceae*	n.d.	-4.18	3.78	0.00	52399
Firmicutes	*Aerococcaceae*	n.d.	-0.36	0.00	0.77	2459569
Firmicutes	*Aerococcaceae*	*Alloiococcus sp*.	-1.81	4.40	-1.59	905303
Firmicutes	*Lactobacillaceae*	*Lactobacillus sp*.	2.33	-0.34	-0.99	4379247
Firmicutes	*Lactobacillaceae*	*Lactobacillus sp*.	0.48	0.00	-0.06	4315658
Firmicutes	*Enterococcaceae*	*Tetragenococcus halophilus*	-3.91	6.13	-1.22	29012
Proteobacteria (α)	*Acetobacteraceae*	*Acetobacter sp*.	1.64	0.00	-0.89	4333237
Proteobacteria (γ)	*Enterobacteriaceae*	*Enterobacter cowanii*	0.20	0.00	0.00	176468
Proteobacteria (γ)	*Enterobacteriaceae*	*Erwinia* sp.	4.54	-1.93	-1.61	1123414
Proteobacteria (γ)	*Enterobacteriaceae*	*Erwinia* sp.	0.24	0.00	0.00	4458775

* OTU Taxonomic assignment using QIIME with Greengenes database

This table shows results of a logistical discrimination analysis that applies numerical values of how predictive OTUs were, to determine which taxa were indicative of each type. The top 19 taxa are represented in this model and their OTU# is given in the far right column. The magnitude of the value in the Dry, Moist, Toombak columns is a measure of relative importance. The sign indicates whether increasing quantities make this OTU more likely (positive sign) or less likely (negative sign) to be predictive of the product type. A zero means that that taxon was not predictive for that type.

The best positive predictors of sample type were OTUs identified as *Tetragenococcus halophilus* (for moist snuff), *Corynebacterium* spp. (for toombak), and *Erwinia* spp. (for dry snuff). Negative predictors indicated that a lack of the *Aerococcaceae* family and *Tetragenococcus halophilus* in dry snuff, and *Staphylococcus succinus* in toombak, were the most predictive for those types of products. The OTUs selected by the prediction model (and the directions of their effects) usually, but not always, correlated well with the patterns of abundances seen in the different product types. For example, two OTUs, associated with the *Corynebacterium* genus, had large positive coefficients for predicting toombak and large negative coefficients for predicting dry or moist snuff, in agreement with the observation that *Corynebacteriaceae* were primarily found in our toombak samples. In some cases, predictors act quantitatively, so that OTUs that are generally present in higher frequency in one product type may be selected, even though these OTUs are found in multiple products. An example is OTU 4312974, which was found in all products but had a generally higher prevalence in moist products than in toombak. This OTU was found to be a positive predictor for moist snuff, but a negative predictor for toombak (Table 2, S4 Table)

### Imputed Metagenomic Survey

The PICRUSt software package [30] was used to infer the potential genetic capability and specific contributions of Bacterial taxa to the imputed metagenome of the tobacco samples. PICRUSt metagenome contributions were computed for all samples, based on KEGG Orthology (KO) terms [31–33]. The PICRUSt output file giving all KO terms is presented in S7 Table. A table of specific OTU contributions to each KO term was also generated, and is presented in S8 Table. A total of 5170 KO terms were identified in the imputed metagenomes in the various products.

**Fig 4:**
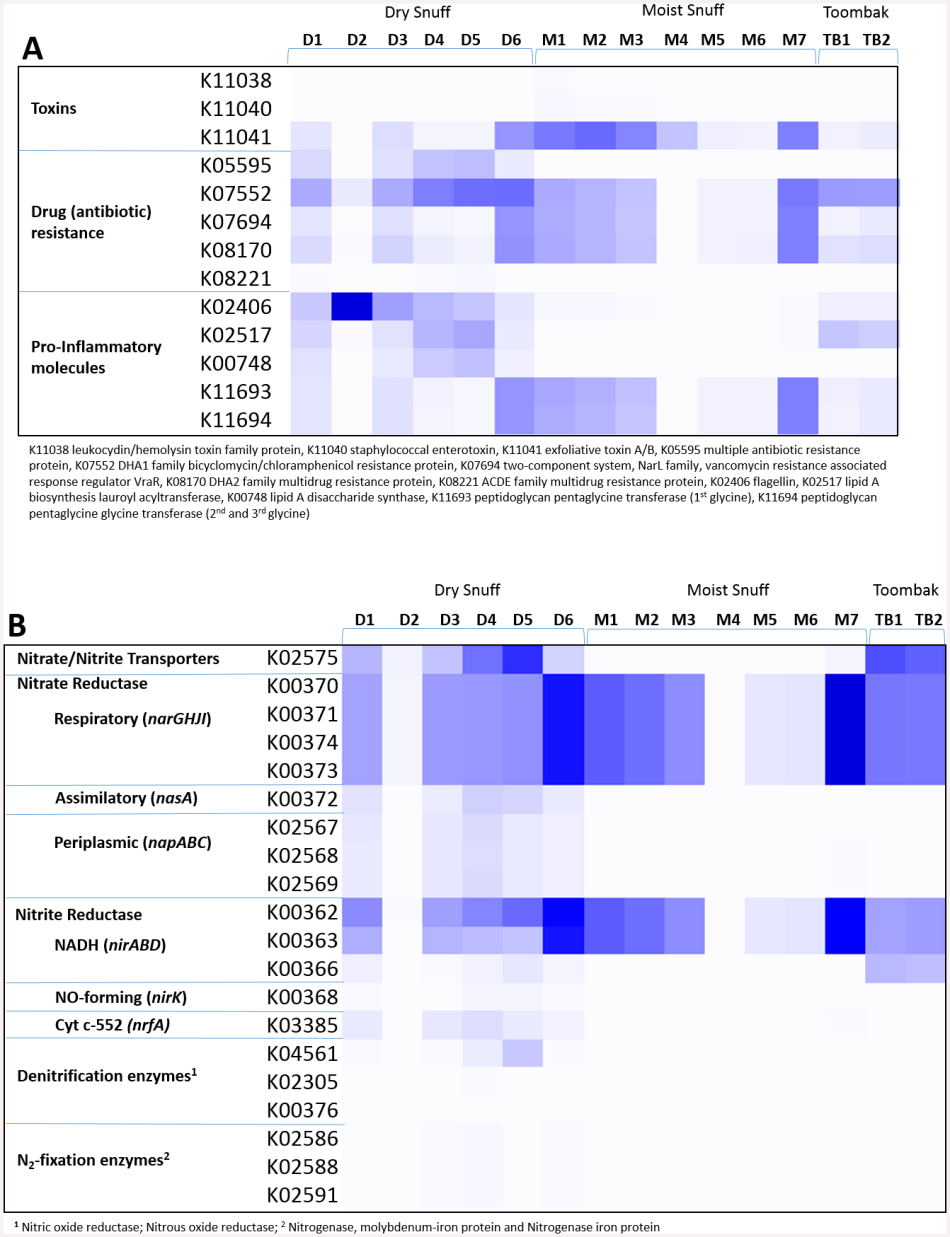
Heatmap displaying key nitrogen metabolism and other genes of interest in smokeless tobacco products. Phylogenetic Investigations of Communities by Reconstruction of Unobserved States (PICRUSt) was used to obtain imputed metagenomic data based on 16S abundances. Displayed here are the predicted relative abundances of genes (grouped by KO terms) per 100,000 reads for **(A)** genes of interest including genes encoding toxins, antibiotic resistance, and pro-inflammatory molecules and **(B)** nitrogen metabolism pathway genes. Respiratory or dissimilatory nitrate reductases (vs. assimilatory) appear to be playing a large role in reduction of nitrate.

Several gene families of interest were investigated (Fig 4A), based on criteria of being both present, and of medical importance, or with the potential for harm in the product (based on pro-inflammatory properties). These included genes encoding for toxins (K11038, K11040 and K11041), antibiotic resistance (K05595, K07552, K07694, K08170, and K08221), and pro-inflammatory molecules, including flagellin (K02406), lipid A (K02517 and K00748), and peptidoglycan (K11693 and K11694).

Given the diversity present in dry snuff, it was not unexpected to find many of these genes present, albeit at various levels in the different products. For dry snuff, the genes investigated further included a toxin gene (K11041), drug resistance genes (mainly K07552, K07694 and K08170), and genes related to flagellin, lipid A and peptidoglycan production. For moist snuff, the genes were more uniform across the seven products. In the moist snuff, several toxin genes (K11041; K11038 and K11040 to a lesser extent), drug resistance genes (mainly K07552 and K08170), and genes related to peptidoglycan production (K11693 and K11694) were observed.

**Fig 5:**
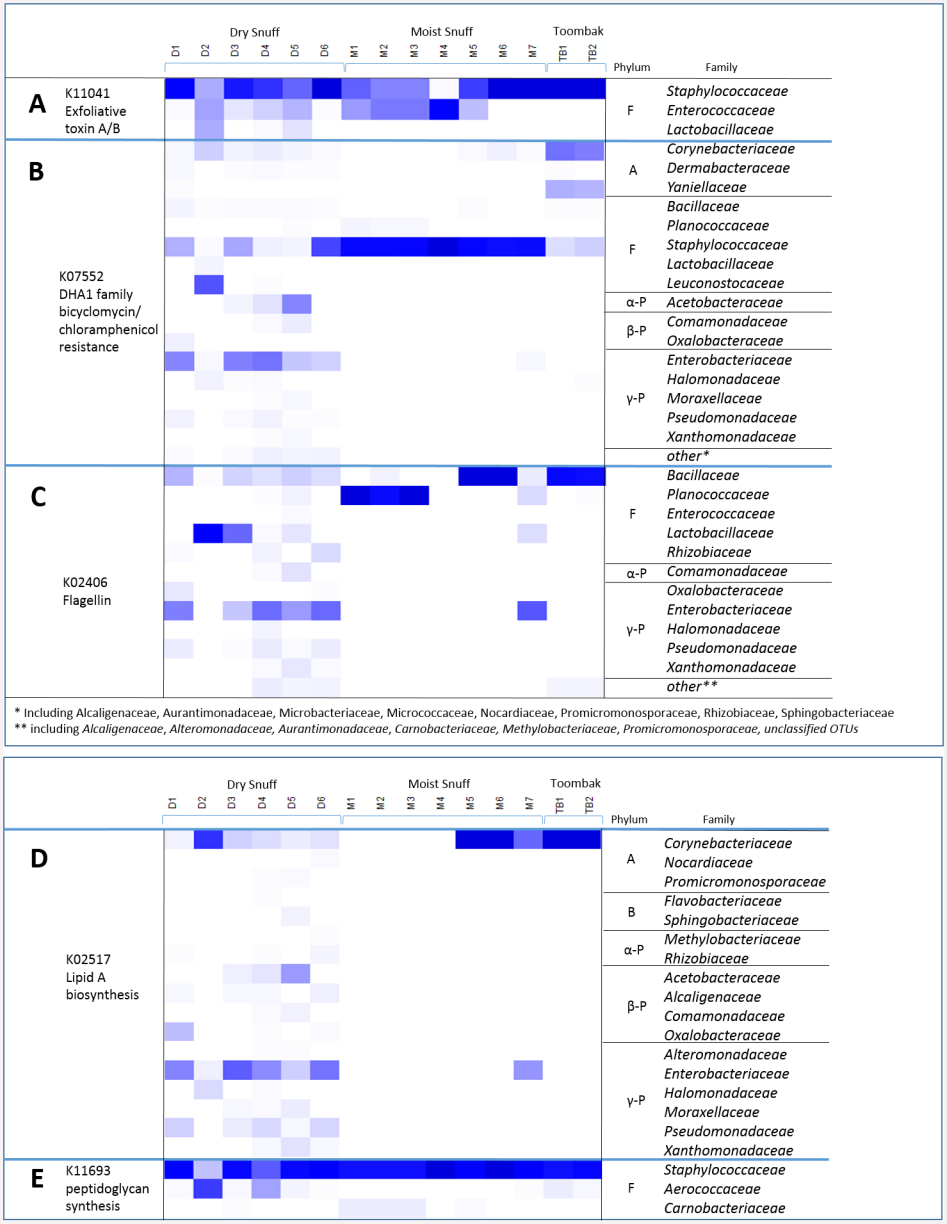
Relative Percentage Contributions for Bacterial Families in Imputed Metagenome for toxin, antibiotic resistance, and pro-inflammatory molecule marker genes. Heatmap representing the percent contributions by bacterial family for [A] K11041 exfoliative toxin A/B, [B] K07552 DHA1 family bicyclomycin/chloramphenicol resistance, [C] K02406 flagellin, [D] K02517 Lipid A biosynthesis, and [E] K11693 peptidoglycan biosynthesis imputed gene abundances. Only contributions above 0.5% are displayed in these heat maps.

A summary of the family-level contributions to some of these genes of interest is found in Fig 5. Only products indicated in Fig 4 to have a certain gene are shown in Fig 5, and even though a single family may represent a high *relative* contribution to a gene in a product, that gene may only be present in a low *absolute* abundance (shown in Fig 4). An example of this in Fig 5B is for family *Staphylococcaceae* in sample M4. Even though *Staphylococcaceae* contributes 100% to the presence of *K07552* in M4, this product had an extremely low abundance of *K07552* compared to other products (too low to be displayed in Fig 4A). Unadjusted numerical representations for gene abundance by OTU are given in S8 Table).

The staphylococcus exfoliative toxin genes (K11041), were mainly predicted to occur in *Staphylococcaceae* for all product types, as expected, but also were predicted in a few other families in the Firmicutes phylum; they were also predicted to occur in *Enterococcaceae* in moist snuff, and in *Enterococcaceae* and *Lactobacillaceae* in dry snuff (Fig 5A).

The presence of antibiotic resistance genes involved in bicyclomycin/chloramphenicol resistance (K07552) appeared primarily among 16 taxonomically diverse families. In dry snuff, these genes had higher predicted prevalence among *Staphylococcaceae* and *Enterobacteriaceae*, and to a lesser extent, *Leuconostocaceae* (D2) and *Acetobacteraceae* (D6). In moist snuff, this KO was predicted to occur primarily in *Staphylcoccaceae*, and with a lower predicted prevalence in *Corynebacteriaceae*, *Halomonadaceae*, and *Planococcaceae*. For toombak, the highest predicted prevalence was in *Corynebacteriaceae*, with a lower predicted prevalence in *Yaniellaceae* and *Staphylococcaceae* (Fig 5B). Genes involved in vancomycin resistance (K07694) were also investigated, and were attributed almost totally (>99%) to the *Staphylococcaceae* family in all products.

Genes encoding flagellin, a key protein involved in bacterial motility and a known pro-inflammatory molecule, were predicted to be found in all products, and were attributed primarily to 11 families of bacteria. The predicted source of flagellin in dry snuff was primarily *Bacillaceae* and *Lactobacillaceae*, *Enterobacteriaceae*, and to a lesser extent, in *Alteromonadaceae* and *Rhizobiacaeae*. The high prevalence prevalence of this gene predicted in product D2 was solely attributed to *Lactobacillaceae*. The predicted source of flagellin in moist snuff was primarily the *Bacillaceae* and *Planococcaceae* families, except for M7, which was also predicted to have flagellin from *Lactobacillaceae* and *Enterobacteriaceae*. The predicted source of flagellin in toombak was almost exclusively in *Bacillaceae* (Fig 5C).

Lipid A synthesis genes, represented by K02517, encode another potentially pro-inflammatory molecule, and were predicted to occur primarily in *Corynebacteriacaeae* among moist snuff and toombak, except M1-M4; one moist snuff (M7) was predicted to have the gene in *Alteromonadaceae*. In dry snuff, the highest sources were predicted to be *Alteromonadaeae*, *Corynebacteriaceae* (especially in D2), *Enterobacteriaceae*, *Halomonadaceae*, and (for D1) *Comamonadaceae* (Fig 5D).

Finally, we also looked at the taxonomic contributions to K11693, encoding one enzyme in the peptidoglycan synthesis pathway. This gene was only found among three families of Firmicutes. The *Staphylococcaceae* family was predicted to be the major source for this gene in all 15 products; toombak additionally was predicted to contain a lesser amount of the gene from *Aerococcaceae* and *Bacillaceae*. Dry snuff was predicted to contain this gene primarily from *Staphylococcaceae* and *Aerococcaceae* (Fig 5E).

The taxonomic metagenomic contributions identified here indicate that many of the toxins and pro-inflammatory molecules were identified in families such as *Staphylococcaceae* and *Enterobacteriaceae*, that are well known to harbor human pathogenic species. This could be an indication that some of these products may actually contain pathogenic species, as was previously found in cigarette tobacco [6], but may also reflect a bias in the database towards human pathogens. Further study into the products themselves and the processing methods is needed to clarify the true abundance of pathogenic species in ST products. A transcriptomic analysis of the products would also be valuable to indicate which species may be active in ST products.

Another pathway of great interest to us was the K.O. Nitrogen Metabolism pathway (PATH:ko00910), containing all annotated nitrate and nitrite reductase and other nitrogen utilization pathway genes (Table 3).

**Table 3:** Key nitrogen metabolism enzymes, TC/EC Number, corresponding microbial genes and their KEGG Orthology numbers.

Nitrate/nitrite transporters	TC number	Gene	KEGG Orthology numbers
Nitrate/H+ symporter (*narK1*) Nitrate/nitrite antiporter (*narK2*)	2.A.1.18.1	*nark*	K02575
*Nitrate/nitrite porter	3.A.1.16.1	*nrtABC*	K15576, K15577, K15578, K15579 *
Nitrate/nitrite transport (*narK* homologs)		*narT*, *narU*	*
**Nitrate Reductases**			
Nitrate Reductases (Respiratory)	1.7.99.4	*narGHJI*	K00370, K00371, K00373, K00374
Nitrate Reductases (Assimilatory)	1.7.99.4	*nasA*	K00372
Nitrate Reductases (Periplasmic)	1.7.99.4	*napAB*	K02567, K02568
**Nitrite reductases**			
Nitrite reductase (NADH)	1.7.1.15	*nirABD*,	K00366, K00362, K00363
Nitrite reductase (cyt c-552)	1.7.2.2	*nrfA*	K03385
Nitrite reductase (ferredoxin)	1.7.7.1	*nirA*	K00366
Nitrite reductase (NO-forming)	1.7.2.1	*nirK*	K00368
**Denitrifying Enzymes**			
Nitric oxide reductase	1.7.2.5	*norBCD*, *norQ*	K04561, K02305
Nitrous oxide reductase	1.7.2.4	*nosZ*	K00376
**Nitrogen Fixation**			
Nitrogenase, molybdenum-iron protein	1.7.2.1	*nifD*, *nifK*	K02586, K02591
Nitrogenase iron protein	1.7.2.5	*nifH*	K02588

* NOTE: *nrt*, *narT and narU* genes were not annotated in this version of KEGG Orthology

During microbial respiration (especially anaerobic), many bacteria use nitrate as an electron acceptor in lieu of oxygen in the maintenance of a proton motive gradient, and in fact, for some it is the preferred electron acceptor in these conditions [36–39]. Respiratory nitrate reductases are often expressed when nitrate is present and O_2_ levels are low [40]; this may account for extracellular nitrite accumulation in oxygen-deprived conditions, such as may exist during fermentation, aging, or storage of tobacco [5,17,19].

Even in microbes with assimilatory pathways, nitrite generated by respiratory processes can be potentially toxic to the microbial cell; therefore, many bacteria contain nitrite exporting enzymes. The presence of nitrate/nitrite anti-porters or nitrite extrusion transporters may contribute to extracellular nitrite levels [37,41]. Extruded nitrite can be further used by microorganisms with assimilatory pathways [42] or dissimilatory (denitrification) pathways [43]. Also, extruded nitrite coupled with favorable conditions (including acidic pH, adequate alkaloid levels) contributes to nitrosation, a chemical process in which nitrite reacts with tobacco alkaloids to form TSNAs [10].

The main contributors to an extracellular accumulation of nitrite are likely those microbes that reduce nitrate to nitrite, and then are able to export it from the cell. Two pathways that are likely to play a role in the generation of extracellular nitrite are encoded by the respiratory (dissimilatory) nitrate reduction *nar* operon, (often containing *narXL*/*narK/narGHJI*, corresponding to regulators/transporter/nitrate reductase) and the periplasmic nitrate reductase *nap* operon [38,44,45]. Both of these pathways generate nitrite from nitrate.

**Fig 6:**
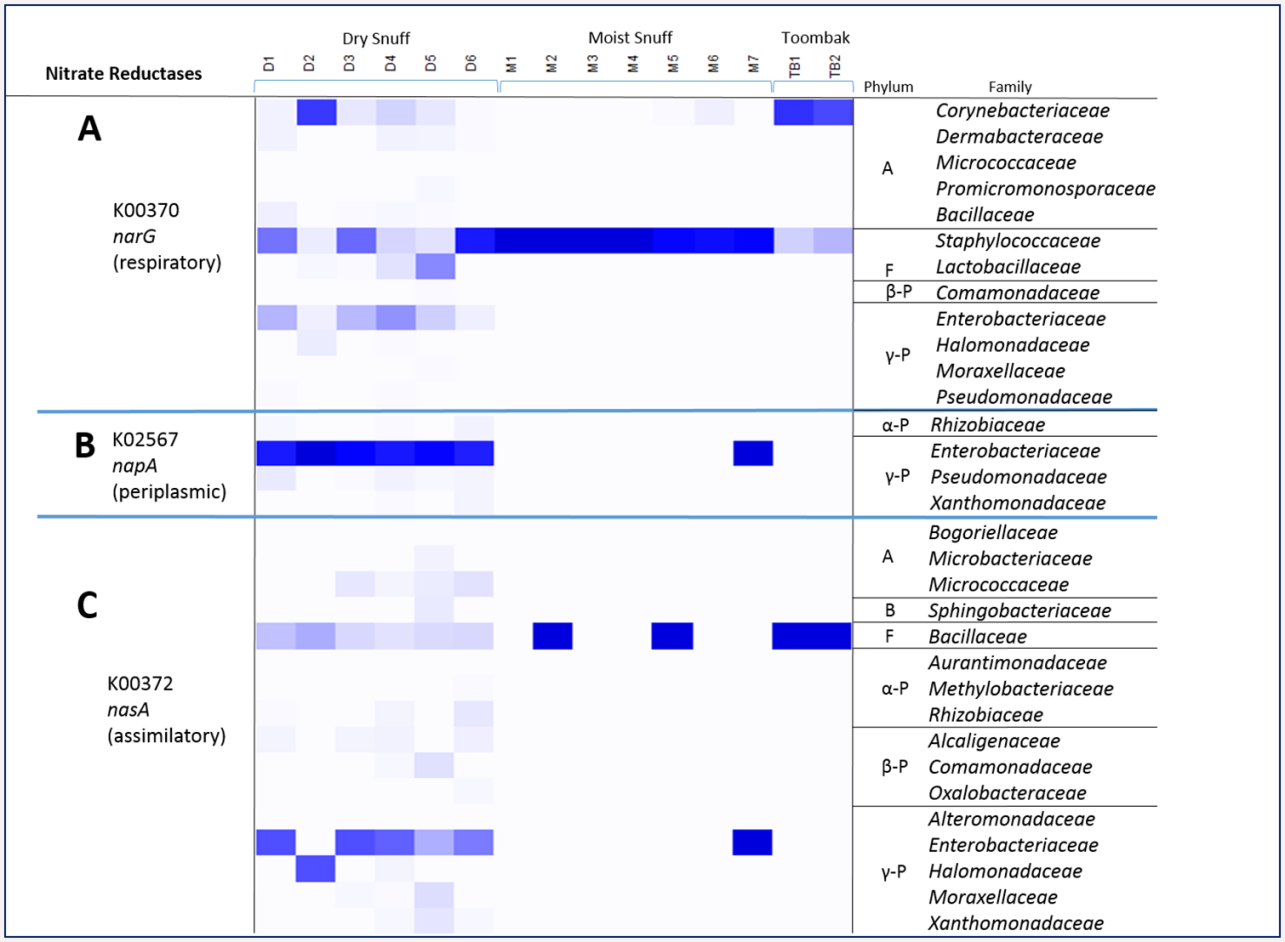
Relative Percentage Contributions for Bacterial Families in Imputed Metagenome for nitrogen metabolism genes. Represented in this heatmap are percentage contributions by bacterial family for [A] respiratory nitrate reductase (*narGHJI*), [B] periplasmic nitrate reductase (*napAB*) and [C] assimitory nitrate reductase (*nasAB*) imputed gene abundances.

A summary of the family-level contributions to the *narG*, *napA*, *and nasA* (representing assimilatory nitrate reductase) genes in the PICRUSt output is found in (Fig 6A, 6B and 6C, **respectively**). As in Fig 5, only products that are indicated in Fig 4 to have a certain gene will have values in Fig 6, and even though a single family may represent a 100% contribution to a gene in a product, that gene may only be present in a very low abundance (shown in Fig 4).

Several routes for reduction of nitrate were identified in the imputed metagenome, with *narGHJI*, *nasA*, and *napAB* predicted to be the three most abundant (Fig 4B). Nitrite reductases were also predicted to be abundant, including *nirBD*, *nirA*, *nirK*, and *nrfA* (Fig 4B). Interestingly, the majority of the contributions to respiratory nitrate reductase (*nar*) were predicted to come from only a few particular OTUs, corresponding primarily to the *Enterobacteriaceae* family and *Corynebacterium*, *Lactobacillus*, and *Staphylococcus* spp. in dry snuff (Fig 6A). In moist snuff, almost all *nar* genes were predicted to come from S*taphylococcus* spp. In toombak, most of the *nar* contributions were predicted to be from *Corynebacterium* and *Staphylococcus* spp.

Based on currently available annotated genomes, members of the *Staphylococcaceae* family generally have at least one copy of *narGHJI*, and some species have *narK*, or a homolog (S5 Table). With the exception of one OTU present in low abundance in the toombak samples, all other OTUs associated with the *Staphylococcaceae* family were assigned to the *Staphylococcus* genus. One OTU (OTU 4312974), assigned the taxonomy of *Staphylococcus succinus*, was present in all 15 products, in considerable amounts in some, and was also predicted to have *nar* genes. *Staphylococcus succinus* and similar coagulase-negative *Staphylococcus* species were recently found to have varying levels of nitrate-reducing capabilities in meat fermentation [46].

Another candidate group that often contains these operons in abundance are members of the family *Enterobacteriaceae*, including the genera *Enterobacter*, *Erwinia*, and *Salmonella*, all of which were identified at the genus level in some products (S6 Table). Members of this family are mainly facultative anaerobes that have *nar* and often the *nap* genes as well.

A third group that may generate extracellular nitrite using the *nar* pathway is the gram-positive genus *Corynebacterium*. All of the OTUs associated with *Corynebacteriaceae* family were assigned to the *Corynebacterium* genus. The species *C*. *ammoniagenes*, *C*. *casei*, *C*. *stationis* (previously *Brevibacterium stationis*) were identified in cigar tobacco products as nitrate reducers [5,47]. Based on currently annotated genomes in IMG, those *Corynebacterium* spp. and others have the *nar* genes (S5 Table). In this study, two OTUs identified as *Corynebacterium stationis* (OTU 650615) and another identified as *Corynebacterium* spp. (OTU 810425) were the OTUs that were most predictive of toombak (Table 2). Those two OTUs along with the one associated with *Staphylococcus succinus* (OTU 4312974) constituted almost all of the *nar* contributions for the two toombak samples (Fig 6A).

Denitrification is a microbial process converting nitrate or other nitrogen-containing small molecules (nitrite, ammonia), to nitrogen gas (N_2_). Based on imputed metagenomic data, five of the six dry snuff and the toombak products were predicted to have denitrification genes present at very low levels, in a few alpha- and beta-proteobacteria (Fig 4B, K04561, K02305, K00376). Only one dry snuff product was predicted to have all three key denitrification genes, but because the predicted incidences were low, this is not likely to be a prominent pathway in that product. Five of the six dry snuff products were predicted to contain nitrogen fixation genes (Fig 4B, K02586, K02588, K02591), albeit at low levels.

Swedish-made snus products are processed using heat treatment to reduce or eliminate microorganisms, resulting in much lower levels of TSNAs than products fermented during processing (e.g. moist snuff, dry snuff, toombak) [12,14–16,48]. If heat treatment is not amenable to domestic production of ST, other means of reducing harmful constituents could be sought. For example, Fisher *et al*. [17] showed that in manufacturing of moist snuff, cleaning fermentation vessels and introducing an excess of non-nitrate-reducing bacteria resulted in lower TSNA levels. Tobacco industry documents also suggest that washing the tobacco leaf surface at the time of harvest helps to reduce or eliminate microbes, soil particles, and agricultural chemicals [18]. Our data suggests that specific groups of bacteria may be contributing to nitrate reduction in these products. If a way could be identified to reduce the prevalence of bacteria in these problematic groups, harmful byproducts in the final products (i.e. nitrite, endotoxins, TSNAs) might be reduced.

One consideration of working with imputed metagenomic data is the reliance on comprehensive database annotation of gene ontology terms. The gene ontology present in the PICRUSt script is based on the Greengenes gg_13_5 database, and accompanying IMG annotation. Although Greengenes is considered a comprehensive 16S Bacterial and Archaeal database, annotation of whole genomes in IMG corresponding to the species in Greengenes is a limiting factor, as is the length and region of 16S sequence used to obtain the initial identifications. Therefore, results using PICRUSt are likely to be biased based on the overall bias in the database (likely to be skewed towards highly studied human pathogenic species). A more accurate imputed metagenome would require better identification of species in the sample (e.g. using longer 16S sequences), more species annotations in the IMG database and finally, the ability to update the scripts to use new annotations added to the database.

Finally, although we focus solely on bacteria in this report, fungi may also contribute to the formation of toxins or carcinogens in ST products, as it is well known that they can play key roles at certain times during the fermentation process [5,49]. A number of fungal species (i.e., *Fusarium*, *Alternaria*, *Candida*, etc.) have been identified in tobacco or tobacco products [5,7,8,49]. Aflatoxin B_1_, which is produced by *Aspergillus* fungi, was recently reported in six U.S.-made dry snuff products (0.01–0.27 μg/g); however, it was not detected among sixteen moist snuff products and three snus products [50]. The presence of other microbes remains to be further explored, using the 18S rDNA or its internal transcribed spacer (ITS) region for analysis, and shotgun metagenomics.

## Conclusions

ST products are highly variable products in terms of tobacco constituents, additives, and processing. Due to the potential harm associated with microbial-driven nitrite production that may result in increased TSNA levels and the presence of pro-inflammatory biomolecules and endotoxins in fermented products, an understanding of microbial influences on tobacco product chemistry has been sought for some time [5,7,17]. Many bacteria have been previously identified in tobacco products (cigarette, cigar, and chewing tobacco). Past studies have used culture-based methods mainly, but some also used molecular approaches [5–7,51]. This manuscript is the first culture-independent survey of bacterial communities focusing on different types of smokeless tobacco.

Among the products analyzed, 33 bacterial families from four phyla, Actinobacteria, Firmicutes, and Proteobacteria and Bacteroidetes, were identified at an abundance of 0.1% or higher. Dry snuff was significantly more diverse than moist snuff. A few core taxonomic groups, such as the *Aerococcaceae* family, *Corynebacterium* and *Staphylococcus* genera, were present at some level in most or all of the products tested.

Relying on imputed metagenomic data, we found that one likely pathway of nitrite generation is the respiratory nitrate reductase pathway, in the *nar* operon gene products. The *nar* operon genes include respiratory nitrate reductase (*narGHJI*) and often contain a nitrate/nitrite antiporter (*narK*) [37,52]. In moist snuff, almost all *nar* contributions were predicted to come from S*taphylococcus* spp.; whereas, in toombak, *nar* genes were predicted to come from *Corynebacterium* and *Staphylococcus* spp. For dry snuff, *nar* genes were predicted to come from the *Corynebacterium*, *Lactobacillus*, and *Staphylococcus* spp. and the *Enterobacteriaceae* family. The *nap* genes that encode periplasmic nitrate reductase, although predicted at lower levels, could also contribute to the accumulation of extracellular nitrite and were predicted predominantly in the *Enterobacteriaceae* family, found in greatest abundance in the dry snuff products.

In addition to the nitrate reducing capability of many bacteria, there are other negative aspects resulting from the presence of bacteria in ST products. These may include the generation and release of toxins and pro-inflammatory molecules, as well as the risk of gene transfer of antibiotic resistance genes from the fermentation and plant-associated species in the product to the user’s oral and/or gastro-intestinal microbiota. Of further concern is the diverse population of bacteria in dry snuff, which may be used orally or inhaled nasally.

This report suggests that a wide array of mostly soil-borne microorganisms are present in typical fermented types of smokeless tobacco. Some of these populations possess nitrate reduction capacity that can contribute to the formation of carcinogenic nitrosamines in ST products [17,53]. Investigations into the microbial communities and their role in tobacco products may shed light on potential means of decreasing nitrite, TSNA levels, or other harmful compounds in ST products. Reducing or eliminating these constituents in ST products should be further pursued and encouraged if technologically practical and feasible.

## Supporting Information

S1 Fig Number of reads per product in split_library_out.txt file. The numbers of (combined replicate) reads for each product at the start of the informatics pipeline are represented as bars.
(TIF)

S2 Fig Alpha Diversity by several metrics. Plots shown represent estimated OTU abundance in increasingly rarefied subsamples (n = 50) of the original data set. Error bars represent standard deviation. Plots were generated in QIIME. (A) Rarefaction plot showing number of observed OTUs (Y-axis) per number of sequences sub-sampled by type, using the observed_species metric (50 points plotted). Where the curves plateau can be considered the place at which more reads do not give more useful data on relative abundances. Our lowest replicate had >11000 sequences, indicating saturation of observed species for all samples. (B) Rarefaction plot showing number of observed OTUs (Y-axis) versus number of sequences sub-sampled (X-axis), by product description, using the PD_whole_tree metric. (C) Rarefaction plot showing number of observed OTUs (Y-axis) versus number of sequences sub-sampled (X-axis), by product description, using the chao1 metric. Error bars represent standard deviation based on 10 random subsamplings.
(TIF)

S3 Fig 2D plots of PCA analysis. 2-dimensional representations of Principal Component Analysis (PCA) by (A) Description and (B) Product Type. Coloring scheme for product type is the same as for panels **B** and **C** of **S4 Fig**.
(TIF)

S1 Table List of primers used in this study. Given here is a list of the primers used to create the 16S amplicons that were used to create the multiplexed DNA libraries that were sequenced. Primers included DNA barcodes designed to permit the demultiplexing of DNA libraries. The barcoded fusion primers were designed to amplify the V4 region of the 16S rDNA.
(DOCX)

S2 Table Sequence metadata: products, raw read #’s, raw Q-scores, read numbers. Numbers of reads were trimmed at various instances throughout the bioinformatics pipeline. This table displays results of some of the steps that were taken to trim and prepare the data for analysis.
(DOCX)

S3 Table Relative Abundance (%) of Bacterial Families in Smokeless Tobacco Products with Abundance Greater than 0.1%. Relative abundance of bacterial families in smokeless tobacco products are given in percentages of each product sampled. These numbers correspond to the bar graphs in Fig 3.
(DOCX)

S4 Table OTU table. This table displays the OTUs and their abundances in the different products. The column on the far right gives the taxonomic classification of the given OTU. Some OTUs were able to be characterized to the species level, and some only to the order or class.
(TSV)

S5 Table Presence of nitrogen utilization genes in annotated genomes of various bacteria. The presence of specific genes was inferred from annotated genomes in the IMG database (US Department of Energy, Joint Genome Institute). Dots represent gene copies and ‘o’ represent predicted homologs. This list was put together to give an overall view at the genus level of nitrogen utilization capabilities of its member species. The presence of a particular species in this list does not indicate it was identified in our study.
(DOCX)

S6 Table OTU table summarized to the genus level of taxonomic classification. Relative abundances are given in percentages. Levels of classification are given before the taxon name as single letters, where p__ is phylum, c__ is class, o__ is order, f__ is family, g__ is genus. Note that not all OTUs are able to be summarized at the genus level, based on the V4 region of the 16S alone.
(TIF)

S7 Table Predicted metagenome by PICRUSt of 15 smokeless tobacco products. This table represents the total abundances of each gene group as represented by K.O. terms. Gene abundances have only been adjusted for 16S copy number by PICRUSt and have not been adjusted for differences in read numbers or abundances in the OTU table (**S7 Table**). This table is from the raw output of the PICRUSt script *predict_metagenome*.*py*.
(TSV)

S8 Table Metagenome Contributions of OTUs to 15 smokeless tobacco products. This table represents the individual contributions of each OTU to the PICRUST metagenome given in S8 Table, adjusted for 16S copy number in the given OTU, as estimated by PICRUSt based on available information in the IMG system. This data has not been adjusted for differences in read numbers or abundances in the OTU table (S7 Table). This table is from the raw output of the PICRUSt script *metagenome_contributions*.*py*.
(TSV)

S9 Table Taxonomy assignments of OTUs based on Greengenes 13_5. This is a tab-delimited table listing taxonomy of each OTU given by seven ranks of taxonomy (Phylum = Rank1, Species = Rank7). Not all OTUs have descriptions at every rank, because the listed taxonomy represents the best estimate based on the 97% identity criteria.
(TSV)

S1 Text Further information about bioinformatics analysis. This text provides further detail about the bioinformatic analysis steps.
(DOCX)
