# Supplementary figures and images for: Characterization of Bacterial Communities in Selected Smokeless Tobacco Products Using 16S rDNA Analysis
Source: PLoS One. 2016 Jan 19;11(1):e0146939. doi: 10.1371/journal.pone.0146939 (PMC4718623; doi:10.1371/journal.pone.0146939)

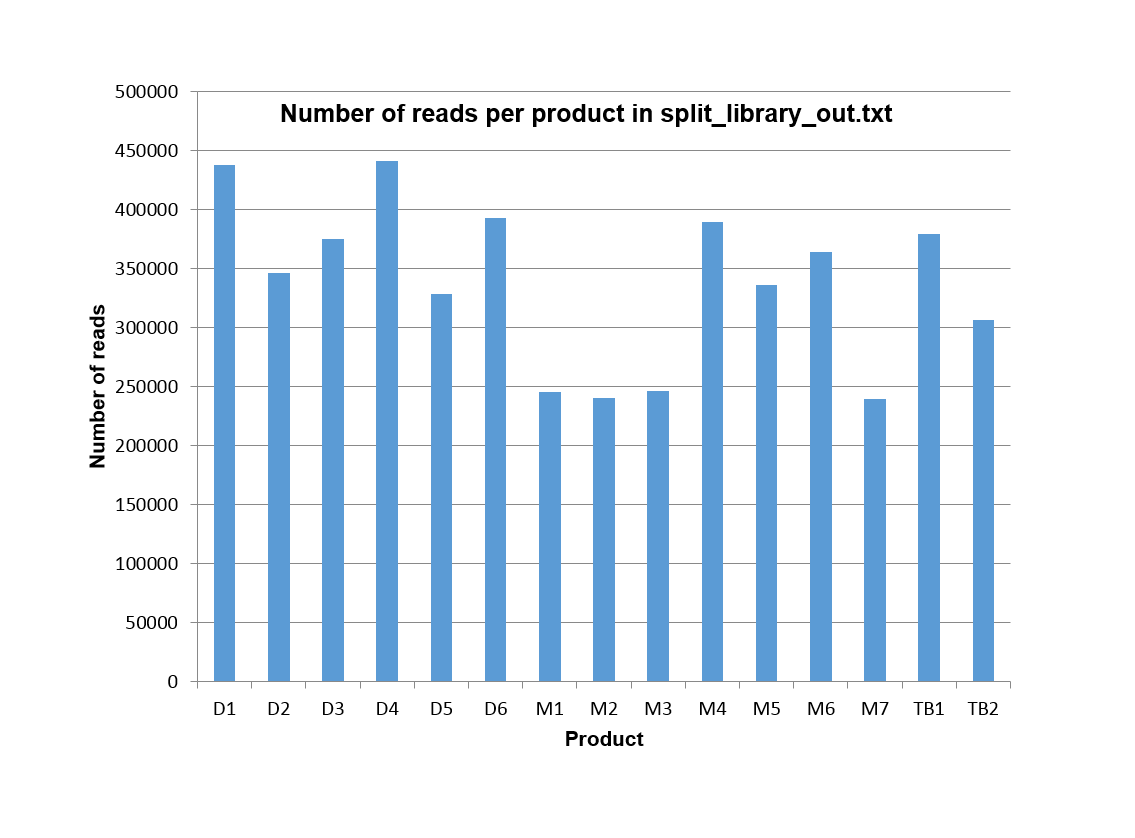

Supplement: S1 Fig — The numbers of (combined replicate) reads for each product at the start of the informatics pipeline are represented as bars. (TIF) [file pone.0146939.s001.tif]

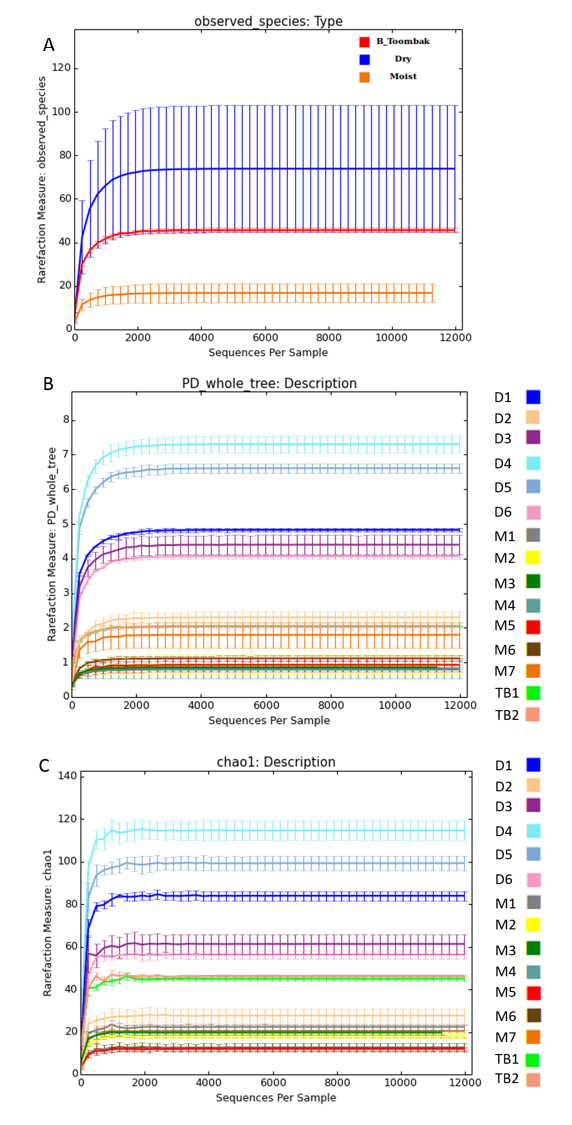

Supplement: S2 Fig — Plots shown represent estimated OTU abundance in increasingly rarefied subsamples (n = 50) of the original data set. Error bars represent standard deviation. Plots were generated in QIIME. (A) Rarefaction plot showing number of observed OTUs (Y-axis) per number of sequences sub-sampled by type, using the observed_species metric (50 points plotted). Where the curves plateau can be considered the place at which more reads do not give more useful data on relative abundances. Our lowest replicate had >11000 sequences, indicating saturation of observed species for all samples. (B) Rarefaction plot showing number of observed OTUs (Y-axis) versus number of sequences sub-sampled (X-axis), by product description, using the PD_whole_tree metric. (C) Rarefaction plot showing number of observed OTUs (Y-axis) versus number of sequences sub-sampled (X-axis), by product description, using the chao1 metric. Error bars represent standard deviation based on 10 random subsamplings. (TIF) [file pone.0146939.s002.tif]

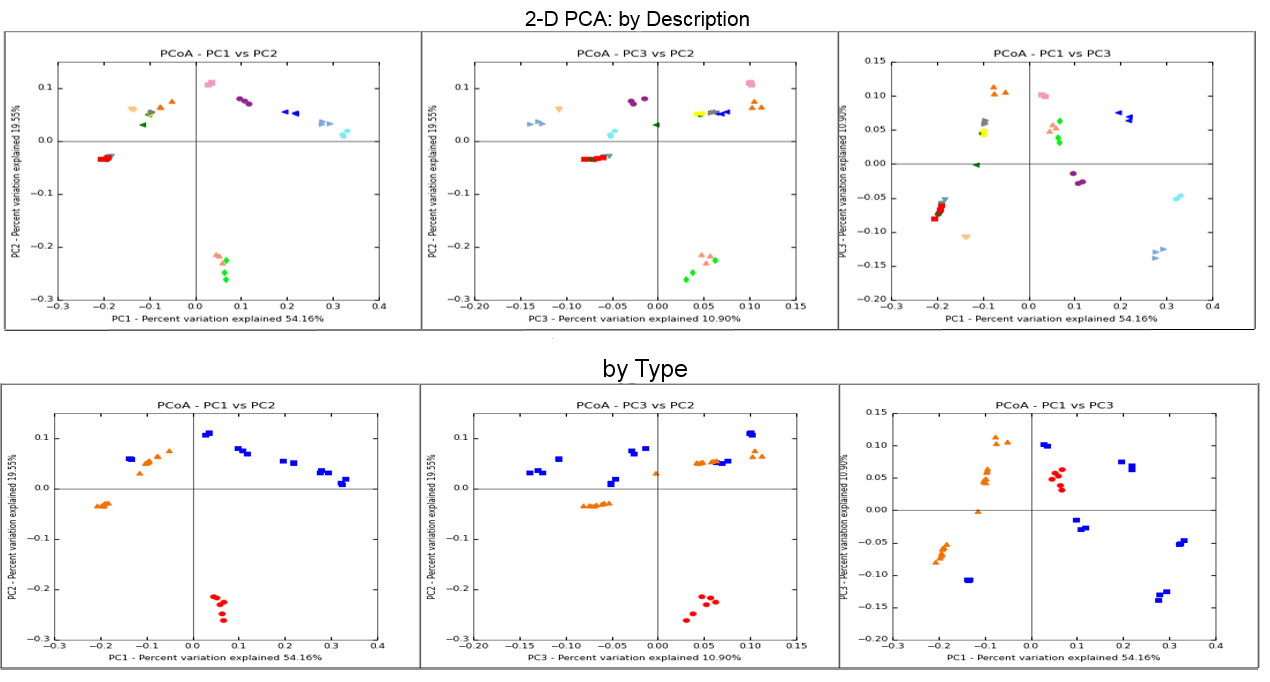

Supplement: S3 Fig — 2-dimensional representations of Principal Component Analysis (PCA) by (A) Description and (B) Product Type. Coloring scheme for product type is the same as for panels B and C of S4 Fig. (TIF) [file pone.0146939.s003.tif]

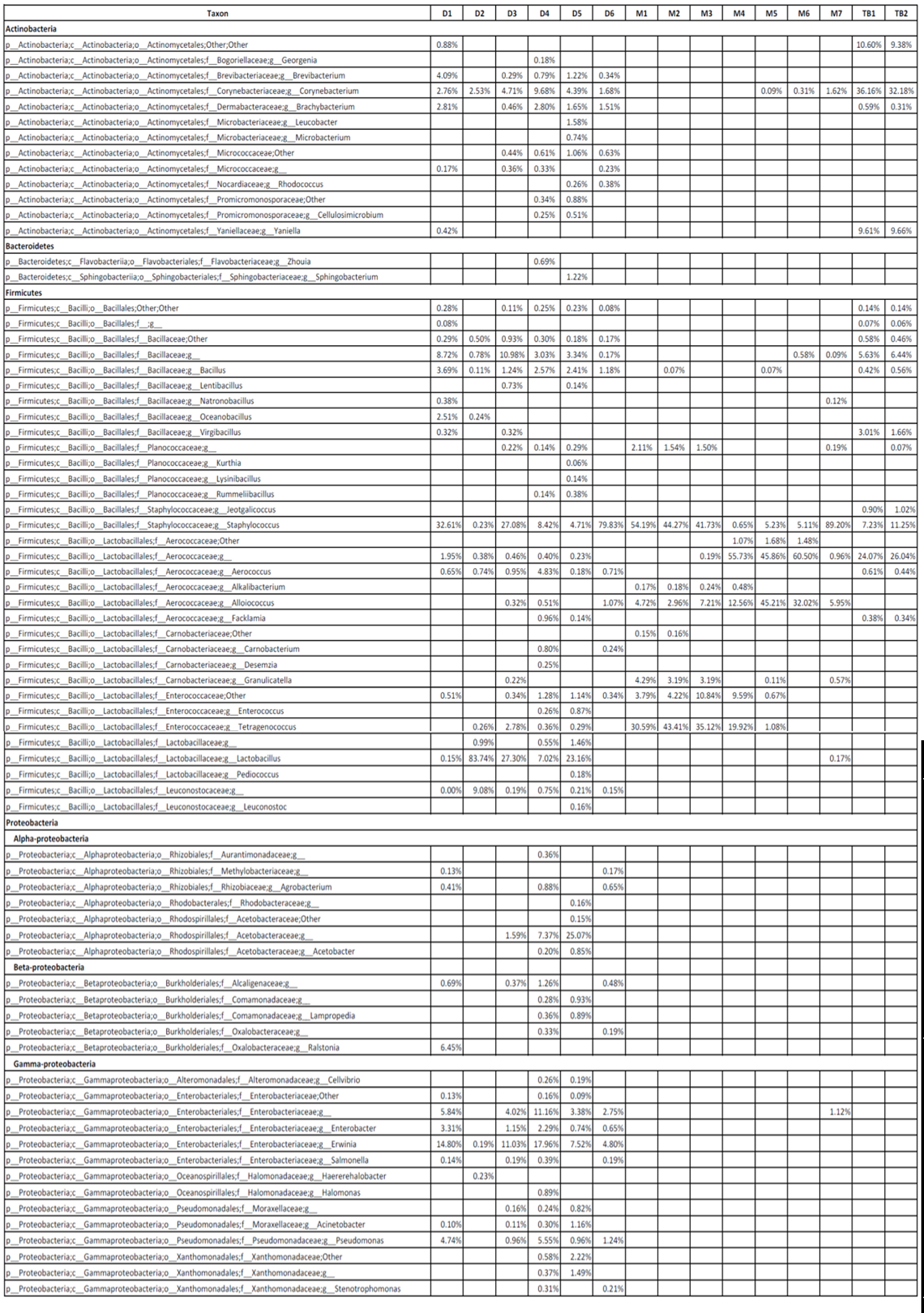

Supplement: S6 Table — Relative abundances are given in percentages. Levels of classification are given before the taxon name as single letters, where p__ is phylum, c__ is class, o__ is order, f__ is family, g__ is genus. Note that not all OTUs are able to be summarized at the genus level, based on the V4 region of the 16S alone. (TIF) [file pone.0146939.s009.tif]
