# Supplementary material for: Characterization of Bacterial Communities in Selected Smokeless Tobacco Products Using 16S rDNA Analysis
Source: PLoS One. 2016 Jan 19;11(1):e0146939. doi: 10.1371/journal.pone.0146939 (PMC4718623; doi:10.1371/journal.pone.0146939)
Supplement: S1 Table — Given here is a list of the primers used to create the 16S amplicons that were used to create the multiplexed DNA libraries that were sequenced. Primers included DNA barcodes designed to permit the demultiplexing of DNA libraries. The barcoded fusion primers were designed to amplify the V4 region of the 16S rDNA. (DOCX) [file pone.0146939.s004.docx]

**S1 Table.** **Primers used in the study**

| **16S Primers** |  |
| --- | --- |
| Sequence Name |  |
| F515 (U515F) | GTGCCAGCMGCCGCGGTAA |
| R806 | GGACTACHVGGGTWTCTAAT |
| trP1 adapter | CCTCTCTATGGGCAGTCGGTGAT |
| A adapter | CCATCTCATCCCTGCGTGTCTCCGAC |
| Key | TCAG |
| **DNA barcodes** |  |
| \| DRY3-1 \| CCGAACACTT \| \| --- \| --- \| \| DRY3-2 \| CTAGGACATT \| \| DRY3-3 \| CTAGGAACCG \| \| DRY1-1 \| CTTCCATAAC \| \| DRY1-2 \| CGGAAGGATG \| \| DRY1-3 \| TTAAGCGGTC \| \| DRY5-1 \| TTGGCTGGAC \| \| DRY5-2 \| CTTGGTTATT \| \| DRY5-3 \| CTTGTCCAAT \| \| DRY4-1 \| CCAGCCTCAA \| \| DRY4-2 \| CTAACCACGG \| \| DRY4-3 \| CGGACAGATC \| \| TOB1-1 \| TTGGCATCTC \| \| TOB1-2 \| TCCTGAATCT \| \| TOB1-3 \| TCCGACAAGC \| \| MST2-1 \| TGAGCGGAAC \| \| MST2-2 \| TAAGGAGAAC \| \| MST2-3 \| TCTAACGGAC \| \| MST6-1 \| TTCGTGATTC \| \| MST6-2 \| TACCAAGATC \| \| MST6-3 \| TAGGTGGTTC \| \| MST5-1 \| AAGAGGATTC \| \| MST5-2 \| CAGAAGGAAC \| \| MST5-3 \| TCCTCGAATC \| \| MST7-1 \| CTGCAAGTTC \| \| MST7-2 \| TTCCGATAAC \| \| MST7-3 \| TCTAGAGGTC \| \| MST4-1 \| CGAAGCGATT \| \| MST4-2 \| CGGAAGAACC \| \| MST4-3 \| TCAGGAATAC \| \| MST1-1 \| CGATCGGTTC \| \| MST1-2 \| TCTGCCTGTC \| \| MST1-3 \| TGAGGCTCCG \| \| MST3-1 \| TTCAATTGGC \| \| MST3-2 \| TCAAGAAGTT \| \| MST3-3 \| TCCTGGCACA \| \| DRY2-1 \| CCTTAGAGTT \| \| DRY2-2 \| TCACTCGGAT \| \| DRY2-3 \| TCCTTGATGT \| \| DRY6-1 \| TCCTAGAACA \| \| DRY6-2 \| TCTGGCAACG \| \| DRY6-3 \| CAGCATTAAT \| \| TOB2-1 \| CCGGAGAATC \| \| TOB2-2 \| CTGGCAATCC \| \| TOB2-3 \| CGGACAATGG \| |  |
| **Fusion Primers** |  |
| F515_Fusion | *trP1 adapter* - **F515** |
| R806_Fusion_BC_X | *A adapter* - Key - Barcode X - **R806** *CCATCTCATCCCTGCGTGTCTCCGAC*TCAGNNNNNNNNNN**GGACTACHVGGGTWTCTAAT** |
