# Supplementary material for: Characterization of Bacterial Communities in Selected Smokeless Tobacco Products Using 16S rDNA Analysis
Source: PLoS One. 2016 Jan 19;11(1):e0146939. doi: 10.1371/journal.pone.0146939 (PMC4718623; doi:10.1371/journal.pone.0146939)
Supplement: S2 Table — Numbers of reads were trimmed at various instances throughout the bioinformatics pipeline. This table displays results of some of the steps that were taken to trim and prepare the data for analysis. (DOCX) [file pone.0146939.s005.docx]

**S2 Table**: Sequence metadata: products, raw read #’s, raw Q-scores, read numbers

| Parameter |  |
| --- | --- |
| Raw Sequences | 8,821,880 |
| Trimmed/Filtered Sequences (fastq_quality_trimmer –l 250 –t 10) | 6,812,005 |
| Sequences discarded due to “barcode errors” (split_libraries.py –b 10 –H 7) | 691,595 |
| “Barcode error” sequences discarded because of barcode not used in analysis | 639,657 |
| Sequences discarded due to primer mismatches | 759,327 |
| Reads discarded due to mean quality score <25 | 246,031 |
| Reads discarded due to Homopolymer > 7 (split_libraries.py –b 10 –H 7) | 36,261 |
| Sequences lengths written into split_library_out (min/max/mean) | 229/557/276.2 |
| Sequence Counts written into split_library_out (min/max/mean) | 20,549/228,067/112,862.02 |
| Sequences written by split_libraries.py | 5,078,791 |
| Sequences in OTU table (reference-picked, gg_13_5) | 3,738,578 |
| Sequences after 0.1% abundance threshold filter | 3,548,180 |
| Sequences lengths in OTU Table mean(standard deviation) | 79,013 (32614) |
| Sequence counts min/max | 11,477/164,828 |
| Sequences lengths in OTU Table, combined replicates, mean(standard deviation) | 236,545 (56,138) |
| Sequence counts, combined replicates, min/max | 163,680/331,857 |
