# Supplementary material for: Characterization of Bacterial Communities in Selected Smokeless Tobacco Products Using 16S rDNA Analysis
Source: PLoS One. 2016 Jan 19;11(1):e0146939. doi: 10.1371/journal.pone.0146939 (PMC4718623; doi:10.1371/journal.pone.0146939)
Supplement: S3 Table — Relative abundance of bacterial families in smokeless tobacco products are given in percentages of each product sampled. These numbers correspond to the bar graphs in Fig 3. (DOCX) [file pone.0146939.s006.docx]

**S3 Table. Relative Abundance (%) of Bacterial Families in Smokeless Tobacco Products with Abundance Greater than 0.1%.**

| **Phylum** |  | **Family** | **D1** | **D2** | **D3** | **D4** | **D5** | **D6** | **M1** | **M2** | **M3** | **M4** | **M5** | **M6** | **M7** | **TB1** | **TB2** |
| --- | --- | --- | --- | --- | --- | --- | --- | --- | --- | --- | --- | --- | --- | --- | --- | --- | --- |
|  | **A** | *Bogoriellaceae ^a^* |  |  |  | 0.2 |  |  |  |  |  |  |  |  |  |  |  |
|  |  | *Brevibacteriaceae ^b^* | 4.1 |  | 0.3 | 0.8 | 1.2 | 0.3 |  |  |  |  |  |  |  |  |  |
|  |  | *Corynebacteriaceae ^c^* | 2.8 | 2.5 | 4.7 | 9.7 | 4.4 | 1.7 |  |  |  |  | 0.1 | 0.3 | 1.6 | **36.2** | **32.2** |
|  |  | *Dermabacteraceae ^d^* | 2.8 |  | 0.5 | 2.8 | 1.6 | 1.5 |  |  |  |  |  |  |  | 0.6 | 0.3 |
| **Actinobacteria** |  | *Microbacteriaceae ^e^* |  |  |  |  | 2.3 |  |  |  |  |  |  |  |  |  |  |
|  |  | *Micrococcaceae ^f^* | 0.2 |  | 0.8 | 0.9 | 1.1 | 0.9 |  |  |  |  |  |  |  |  |  |
|  |  | *Nocardiaceae ^g^* |  |  |  |  | 0.3 | 0.4 |  |  |  |  |  |  |  |  |  |
|  |  | *Promicromonosporaceae ^h^* |  |  |  | 0.6 | 1.4 |  |  |  |  |  |  |  |  |  |  |
|  |  | *Yaniellaceae ^i^* | 0.4 |  |  |  |  |  |  |  |  |  |  |  |  | 9.6 | 9.7 |
| **Firmicutes** | **B** | *Bacillaceae ^j^* | **15.9** | 1.6 | **14.2** | 5.9 | 6.1 | 1.5 |  | 0.1 |  |  | 0.1 | 0.6 | 0.2 | 9.6 | 9.1 |
|  |  | *Planococcaceae ^k^* |  |  | 0.2 | 0.3 | 0.9 |  | 2.1 | 1.5 | 1.5 |  |  |  | 0.2 |  | 0.1 |
|  |  | *Staphylococcaceae ^l^* | **32.6** | 0.2 | **27.1** | 8.4 | 4.7 | **79.8** | **54.2** | **44.3** | **41.7** | 0.7 | 5.2 | 5.1 | **89.2** | 8.1 | **12.3** |
|  | **L** | *Aerococcaceae ^m^* | 2.6 | 1.1 | 1.7 | 6.7 | 0.6 | 1.8 | 4.9 | 3.1 | 7.6 | **69.8** | **92.7** | **94** | 6.9 | **25.1** | **26.8** |
|  |  | *Carnobacteriaceae ^n^* |  |  | 0.2 | 1.1 |  | 0.2 | 4.4 | 3.3 | 3.2 |  | 0.1 |  | 0.6 |  |  |
|  |  | *Enterococcaceae ^o^* | 0.5 | 0.3 | 3.1 | 1.9 | 2.3 | 0.3 | **34.4** | **47.6** | **46** | **29.5** | 1.8 |  |  |  |  |
|  |  | *Lactobacillaceae ^p^* | 0.1 | **84.7** | **27.3** | 7.6 | **24.8** |  |  |  |  |  |  |  | 0.2 |  |  |
|  |  | *Leuconostocaceae ^q^* |  | 9.1 | 0.2 | 0.7 | 0.4 | 0.2 |  |  |  |  |  |  |  |  |  |
| **Bacteroides** | **F** | *Flavobacteriaceae ^r^* |  |  | 0.7 |  |  |  |  |  |  |  |  |  |  |  |  |
|  | **S** | *Sphingobacteriaceae ^s^* |  |  |  | 1.2 |  |  |  |  |  |  |  |  |  |  |  |
|  | **α** | *Acetobacteraceae ^t^* |  |  | 1.6 | 7.6 | **26.1** |  |  |  |  |  |  |  |  |  |  |
|  |  | *Aurantimonadaceae ^f^* |  |  |  | 0.4 |  |  |  |  |  |  |  |  |  |  |  |
|  |  | *Methylobacteriaceae ^f^* | 0.1 |  |  |  |  | 0.2 |  |  |  |  |  |  |  |  |  |
|  |  | *Rhizobiaceae ^f^* | 0.4 |  |  | 0.9 |  | 0.7 |  |  |  |  |  |  |  |  |  |
|  |  | *Rhodobacteraceae ^f^* |  |  |  |  | 0.2 |  |  |  |  |  |  |  |  |  |  |
|  | **β** | *Alcaligenaceae ^f^* | 0.7 |  | 0.4 | 1.3 |  | 0.5 |  |  |  |  |  |  |  |  |  |
|  |  | *Comamonadaceae ^u^* |  |  |  | 0.6 | 1.8 |  |  |  |  |  |  |  |  |  |  |
| **Proteobacteria** |  | *Oxalobacteraceae ^v^* | 6.5 |  |  | 0.3 |  | 0.2 |  |  |  |  |  |  |  |  |  |
|  | **γ** | *Alteromonadaceae ^w^* |  |  |  | 0.3 | 0.2 |  |  |  |  |  |  |  |  |  |  |
|  |  | *Enterobacteriaceae ^x^* | **24.2** | 0.2 | **16.4** | **32.0** | **11.7** | 8.4 |  |  |  |  |  |  | 1.1 |  |  |
|  |  | *Halomonadaceae ^y^* |  | 0.2 |  | 0.9 |  |  |  |  |  |  |  |  |  |  |  |
|  |  | *Moraxellaceae ^z^* | 0.1 |  | 0.3 | 0.5 | 2.0 |  |  |  |  |  |  |  |  |  |  |
|  |  | *Pseudomonadaceae ^aa^* | 4.7 |  | 1.0 | 5.5 | 1.0 | 1.2 |  |  |  |  |  |  |  |  |  |
|  |  | *Xanthomonadaceae ^bb^* |  |  |  | 1.3 | 3.7 | 0.2 |  |  |  |  |  |  |  |  |  |
| **Other (Incl. unidentified)** *^cc^* |  |  | 1.3 | 0.1 | 0 | 0.1 | 0 | 0 | 0 | 0.1 | 0 | 0 | 0 | 0 | 0 | 10.8 | 9.5 |
| **Total (%)** |  |  | 100 | 100 | 100 | 100 | 100 | 100 | 100 | 100 | 100 | 100 | 100 | 100 | 100 | 100 | 100 |

Bacterial Orders: A = Actinomycetalesr; B= Bacillales, L=Lactobacillales; F=Flavobacteriales; S=Sphingobacteriales

*^a^ Bogoriellaceae* only included the *Georgenia* genus (1 OTU)
*^b^ Brevibacteriaceae* only included the *Brevibacterium* genus (5 OTUs) *^c^ Corynebacteriaceae* only included the *Corynebacterium* genus (11 OTUs) *^d^ Dermabacteraceae* only included the *Brachybacterium* genus (2 OTUs) *^e^* *Microbacteriaceae* only included the *Leucobacter* (1 OTU) and *Microbacterium* genera (1 OTU) *^f^* genus not specified *^g^* *Nocardiaceae* only included the *Rhodococcus* genus (2 OTUs) *^h^ Promicromonosporaceae* only included *Cellulosimicrobium* genus (1 OTU) *^i^* *Yaniellaceae* only included the *Yaniella* genus (2 OTUs) *^j^* *Bacillaceae* included the following genera: *Bacillus* (13 OTUs)*, Lentibacillus* (1 OTU)*, Natronobacillus* (1 OTU)*, Oceanobacillus* (4 OTUs)*, and Virgibacillus* (7 OTUs). _28 OTUs were undesignated at the genus level. *^k^* *Planococceceae* included *Kurthia* (1 OTU), *Lysinibacillus* (1 OTU), and *Rummeliibacillus* (1 OTU) genera; 5 OTUs were undesignated that the genus level. *^l^* *Staphylococcaceae* included the *Staphylococcus* genus (13 OTUs), except for 1 OTU designated as *Jeotgalicoccus* (present at low levels in both toombak samples). *^m^* *Aerococcaceae* included the following genera: *Aerococcus*, *Alkalibacterium*, *Alloiococcus,* and *Facklamia* (1 OTU each); 10 OTUs were undesignated at the genus level. *^n^ Carnobacteriaceae* included the following genera: *Carnobacterium* (1 OTU), *Desemzia* (1 OTU), and *Granulicatella* (3 OTU); 1 OTU was undesignated at genus level. *^o^* *Enterococcaceae* included *Tetragenococcus* (3 OTUs) and *Enterococcus* (2 OTUs) genera; 3 OTUs were undesignated at the genus level. *^p^* *Lactobacillaceae* included mainly *Lactobacillus* (18 OTUs) with a single OTU designated as *Pediococcus* (1 OTU) found in low abundance in D5 only. 2 OTUs were _undesignated at the genus level in a few dry snuffs. *^q^ Leuconostocaceae* included *Leuconostoc* genus (1 OTU) and 4 OTUs undesignated at the genus level. *^r^ Flavobacteriaceae* included only the *Zhouia* genus (1 OTU) *^s^ Sphingobacteriaceae* included only the *Sphingobacterium* genus *(*5 OTUs) *^t^ Acetobacteraceae* included the *Acetobacter* genus (2 OTU) and 2 OTUs undesignated at the genus level. *^u^ Comamonadaceae* included the *Lampropedia* genus (1 OTU) and 1 OTU undesignated at the genus level. *^v^ Oxalobacteraceae* included the *Ralstonia* genus (1 OTU) and 1 OTU undesignated at the genus level. *^w^ Alteromonadaceae* included only the *Cellvibrio* genus (2 OTUs) *^x^* *Enterobacteriaceae* included *Enterobacter* (1 OTU), *Erwinia* (4 OTU), and *Salmonella* (1 OTU) genera; 17 OTUs were undesignated at the genus level. *^y^ Halomonadaceae* included *Haererehalobacter* (1 OTU) and *Halomonas* (3 OTUs) genera. *^z^* *Moraxellaceae* included *Acinetobacter* genus (4 OTUs); 1 OTU were undesignated at the genus level. *^aa^ Pseudomonadaceae* only included the *Pseudomonas* genus (10 OTUs) *^bb^ Xanthomonadaceae* included the *Stenotrophomonas* genus (1 OTU); 3 OTUs were undesignated at the genus level. *^cc^* These precentages include all OTUs only designed to the order level.
