# Supplementary material for: Characterization of Bacterial Communities in Selected Smokeless Tobacco Products Using 16S rDNA Analysis
Source: PLoS One. 2016 Jan 19;11(1):e0146939. doi: 10.1371/journal.pone.0146939 (PMC4718623; doi:10.1371/journal.pone.0146939)
Supplement: S5 Table — The presence of specific genes was inferred from annotated genomes in the IMG database (US Department of Energy, Joint Genome Institute). Dots represent gene copies and ‘o’ represent predicted homologs. This list was put together to give an overall view at the genus level of nitrogen utilization capabilities of its member species. The presence of a particular species in this list does not indicate it was identified in our study. (DOCX) [file pone.0146939.s008.docx]

| **Family** | | **Genus/species** | | ***Nitrate/Nitrite Transporters*** | | ***Nitrate Reductases*** | | | ***Nitrite Reductase*** |
| --- | --- | --- | --- | --- | --- | --- | --- | --- | --- |
|  | |  | | ***nrtABC*** | ***narK*** | ***narGHIJ*** | ***napAB*** | ***nasAB*** | ***nirBD*** |
| **Actinobacteria (gram +)** | | | |  |  |  |  |  |  |
| C | | *Corynebacterium* | *ammoniagenes* | **⚫** | **⚫** | **⚫** |  |  |  |
| C | | *Corynebacterium* | *casei* | **⚫⚫⚫** | **⚫** | **⚫** |  |  |  |
| C | | *Corynebacterium* | *durum* | **⚫** | **⚫** | **⚫** |  |  |  |
| C | | *Corynebacterium* | *doosanense* | **⚫** | **⚫** | **⚫** |  |  |  |
| C | | *Corynebacterium* | *efficiens* | **⚫** | **⚫⚫** | **⚫** |  |  |  |
| C | | *Corynebacterium* | *glutamicum* | **⚫** | **⚫** | **⚫** |  |  |  |
| C | | *Corynebacterium* | *halotolerans* | **⚫** | **⚫** | **⚫** |  |  | **⚫** |
| C | | *Corynebacterium* | *maris* | **⚫** | **⚫** | **⚫** |  |  |  |
| D | | *Brachybacterium* | *faecium* |  | **⚫** | **⚫** |  |  | **⚫** |
| **Firmicutes (gram +)** | | | |  |  |  |  |  |  |
| B | | *Bacillus* | *clausii* |  | **⚫⚫** | **⚫** |  |  | **⚫** |
| B | | *Bacillus* | *subtilis* |  | **⚫⚫** | **⚫** |  |  | **⚫** |
| **B** | | *Virgibacillus* | *halodenitrificans* |  | **⚫** | **⚫** |  |  |  |
| LA | | *Lactobacillus* | *oris* |  | **⚫** | **⚫** |  |  |  |
| S | | *Staphylococcus* | *epidermidis* |  | **⭘** | **⚫** |  |  | **⚫** |
| S | | *Staphylococcus* | *equorum* |  | **⭘** | **⚫** |  |  | **⚫** |
| S | | *Staphylococcus* | *kloosii* |  | **⭘** | **⚫** |  |  | **⚫** |
| S | | *Staphylococcus* | *pasteuri* |  | **⭘** | **⚫** |  |  | **⚫** |
| S | | *Staphylococcus* | *saprophyticus* |  | **⭘** | **⚫** |  |  | **⚫** |
| S | | *Staphylococcus* | *sciuri* |  | **⭘** | **⚫** |  |  | **⚫** |
| S | | *Staphylococcus* | *succinus* |  | **⭘** | **⚫** |  |  | **⚫** |
| Family: C=Corynebacteriaceae; D=Bacillaceae; LA=Lactobacillaceae; S=Staphylococcaceae   \|  \| \| --- \| \|  \|  \| **Family** \| **Genus/species** \| \| ***Nitrate/Nitrite Transporters*** \| \| ***Nitrate Reductases*** \| \| \| ***Nitrite Reductase*** \| \| --- \| --- \| --- \| --- \| --- \| --- \| --- \| --- \| --- \| \|  \|  \|  \| ***nrtABC*** \| ***narK*** \| ***narGHIJ*** \| ***napAB*** \| ***nasAB*** \| ***nirBD*** \| \| **Alphaproteobacteria (gram -)** \| \| \|  \|  \|  \|  \|  \|  \| \| AC \| *Acetobacter* \| *pasteurianus* \|  \| **⚫** \| **⚫^1^** \|  \|  \|  \| \| **Betaproteobacteria (gram -)** \| \| \|  \|  \|  \|  \|  \|  \| \| *OX* \| *Ralstonia* \| *solanacearum* \| **⚫** \| **⚫⚫⚫** \| **⚫** \| **⚫** \|  \| **⚫** \| \| ***Gammaproteobacteria (gram -)*** \| \| \|  \|  \|  \|  \|  \|  \| \| *EB* \| *Citrobacter* \| *amalonaticus* \|  \| **⚫⚫** \| **⚫⚫** \| **⚫** \|  \| **⚫** \| \| EB \| *Cronobacter* \| *muytjensi* \| **⚫** \| **⚫** \| **⚫** \|  \|  \| **⚫** \| \| EB \| *Enterobacter* \| *absuriae* \| **⚫** \| **⚫** \| **⚫** \|  \| **⚫** \| **⚫** \| \| EB \| *Enterobacter* \| *hormaechei* \| **⚫** \| **⚫** \| **⚫** \|  \| **⚫** \| **⚫** \| \| EB \| *Enterobacter* \| *cloacae* \| **⚫** \| **⚫** \| **⚫** \|  \|  \| **⚫** \| \| EB \| *Escherichia* \| *coli* \|  \| **⚫** \| **⚫** \| **⚫** \|  \| **⚫** \| \| EB \| *Pantoea* \| *agglomerans* \| **⚫** \| **⚫** \| **⚫** \|  \| **⚫** \| **⚫*** \| \| EB \| *Pectobacterium* \| *cartovorum* \| **⚫** \| **⚫** \| **⚫** \| **⚫** \| **⚫** \| **⚫** \| \| EB \| *Salmonella* \| *enterica (arizonae)* \|  \| **⚫** \| **⚫** \| **⚫** \|  \| **⚫** \| \| H \| *Halomonas* \| *cupida* \|  \| **⚫⚫⚫** \| **⚫⚫** \|  \| **⚫** \| **⚫** \| \| H \| *Halomonas* \| *campaniensis* \|  \| **⚫⚫⚫** \| **⚫** \|  \|  \| **⚫** \| \| ME \| *Methylobacterium* \| *radiotolerans* \|  \| **⚫⚫⚫** \| **⚫** \|  \|  \|  \| \| ME \| *Methylobacterium* \| *nodulans* \|  \| **⚫⚫⚫** \| **⚫** \|  \|  \|  \| \| Family: AC=Acetobacteraceae; OX=Oxalobacteraceae; EB=Enterobacteriaceae; H=Halomonadaceae; ME= Methylobacteriaceae \| \| \| \| \| \| \| \| \| \| \| \|  \| \| \| \| \| \| \| \| \| \| \| | | | | | | | | | |
|  | | | | | | | | | |

**^1^** Only annotated for 1 of the 3 strains presently in the IMG database (<http://img.jgi.doe.gov/>) accessed June 25, 2015
