# Supplementary material for: Characterization of Bacterial Communities in Selected Smokeless Tobacco Products Using 16S rDNA Analysis
Source: PLoS One. 2016 Jan 19;11(1):e0146939. doi: 10.1371/journal.pone.0146939 (PMC4718623; doi:10.1371/journal.pone.0146939)
Supplement: S1 Text — This text provides further detail about the bioinformatic analysis steps. (DOCX) [file pone.0146939.s013.docx]

**Supplemental Information Text: Bioinformatics**

All data was processed on computers running Biolinux versions 7 or 8 [1]. Fastx_toolkit (from the Gregory J. Hannon Lab, Cold Spring Harbor, NY, <http://cancan.cshl.edu/labmembers/gordon/fastx_toolkit/index.html>) was used to remove the sequences less than 250 base pairs, using the *fastq_quality_trimmer* script with parameters –l 250 –t 10 (remove sequences shorter than 250 base pairs and less than Q-score of 10). The remaining 6,812,005 sequences were used for further analysis. Raw sequence files from three sequencing runs were catenated into a single fastq. Sequence names in the raw sequence files were changed to protect identity of products. The fastq file was converted to a fasta and .qual file using the QIIME python script *convert_fastaqual_fastq.py*, using the –c fastq_to_fastaqual parameter. Demultiplexing was handled using the QIIME script split_libraries.py with the parameters –b 10 –H 7 (10 base pair barcode, remove homopolymers greater than 7 base pairs). In the data set, 691,595 sequences were removed due to barcode errors (of which 639,657, representing 92.5% of these, were from a sample that was not analyzed), 759,327 sequences were removed due to primer mismatches, and 36,261 sequences were removed due to homopolymer length greater than 7 (**S2 Table**). Total sequences written by *split_libraries.py* script were 5078791 (**S1 Figure**).

OTUs were picked using closed-reference picking with the Uclust algorithm through the QIIME script *parallel_pick_otus_uclust_ref.py* using parameters –z –O 6 (reverse strand checking with 6 processes) with the Greengenes 13_5 97_otus.fasta as the reference set [2]. A representative set was chosen using Uclust with the QIIME script *pick_rep_set.py* also using the Greengenes 13_5 97_otus.fasta reference set. Taxonomy was assigned based on the Greengenes reference taxonomy for 97% identity. The initial OTU table contained 4,209,720 reads in 5424 OTUs. OTUs ascribed to chloroplast and mitochondria (50/5424, containing 462,086 reads in pre-trimmed OTU table) were pooled and removed from the representative set. Sequences were then aligned using the pynast algorithm and filtered using the QIIME scripts: *align_seqs.py* and *filter_alignment.py* [3]. OTUs that failed to align (29 OTUs) were removed from the representative set. OTUs that failed alignment, as well as chloroplast and mitochondria OTUs, were excluded from the OTU table, which was constructed using the QIIME *make_otu_table.py* script. A tree was built using the *make_phyogeny.py* QIIME script. A tree file was built using the *make_phyogeny.py* QIIME script

After filtering and removing reads associated with OTUs corresponding to chloroplast and mitochondria, removing OTUs that failed alignment, and applying a 0.1% abundance threshold, the average number of reads obtained per sample in the OTU table was 79013 reads (standard deviation 32614), with a minimum of 11477 and 164828. The abundance threshold was used for purposes of removing spurious OTUs due to sequencing errors, as well as for facilitating statistical analysis of the most abundant OTUs. Overall abundance was graphed by first pooling all replicates into samples and normalizing for 16S gene copy number (using PICRUSt script *normalize_by_copy­_number.py*). The R script used in the 0.1% cutoff, threshold.matrix.R, is available for download on github: <https://github.com/CDC-DLS-TVB/R-scripts>.

Tables were inter-converted between tab-separated variable files and .biom files to facilitate sorting [4]. Taxonomy was added to the .biom files during OTU table generation, and taxonomic tree files, alpha-rarefaction curves, and taxa rank abundance summaries were generated using QIIME. Heatmaps were generated in R using gplots package [5].

PICRUSt scripts were used to obtain imputed metagenome data. The script predict_metagenomes.py was implemented using the Greengenes 13_5 reference database. Finally, contributions by taxa were calculated using the PICRUSt script *metagenome_contributions.py*. KO terms relating to genes of interest, including the Nitrogen metabolism (KO pathway ko00910), were parsed from the main predictions and contributions output tables, adjusted for total abundance based on the OTU table, and analyzed.

PICRUSt output was summarized to the family level by providing tab-delimited files of individual genes from PICRUSt into a custom R script, *metagenome_family_contributions.R*. The output from this was formatted in Microsoft Excel 2013 into a tab-delimited table and run through another custom R script, *generate_family_heatmap.R* (R scripts are available at <https://github.com/CDC-DLS-TVB/R-scripts>).

1. Field D, Tiwari B, Booth T, Houten S, Swan D, Bertrand N and Thurston M (2006) Open Software for biologists: from famine to feast. Nature Biotechnology 24: 801 – 803.
2. Edgar RC (2010) Search and clustering orders of magnitude faster than BLAST, Bioinformatics 26(19): 2460-2461.
3. Caporaso JG, Bittinger K, Bushman FD, DeSantis TZ, Andersen GL, Knight R (2010) PyNAST: a flexible tool for aligning sequences to a template alignment. Bioinformatics 26(2): 266-267.
4. McDonald D, Clemente JC, Kuczynski J, Rideout JR, Stombaugh J, Wendel D, et al. (2012) GigaScience 1:7. doi:10.1186/2047-217X-1-7.
5. Warnes GR, Bolker B, Bonebakker L, Gentleman R, Liaw WHA, Lumley T, et al*.* (2015) gplots: Various R Programming Tools for Plotting Data. https://cran.r-project.org/web/packages/gplots/index.html
